# Molecular, Crystal, and Surface Chemistry of *p*‑Aminobenzoic Acid and the Interrelationship between Its Solution-Phase Crystallization and Solid-Form Selectivity: A Review

**DOI:** 10.1021/acs.cgd.5c01190

**Published:** 2025-10-29

**Authors:** Cai Y. Ma, Kevin J. Roberts

**Affiliations:** Centre for the Digital Design of Drug Products, School of Chemical and Process Engineering, 4468 University of Leeds, Woodhouse Lane, Leeds LS2 9JT, U.K.

## Abstract

An overview of some selective research into the solution-phase crystallization and polymorphic behavior of the organic compound, *p*-aminobenzoic acid (PABA), a representative model compound for a small-molecule pharmaceutical, is presented. The review highlights and interlinks the crystal science underpinning its molecular properties, crystallographic structures, intermolecular crystal chemistry, solution structuring properties, crystallizability, nucleation and growth kinetics and mechanisms, polymorph selection and solvent-mediated morphology, and surface chemistry using both computational modeling and experimental studies. The review mostly focuses on studies of the formation of the α-polymorphic form and how solvent selection and additive action can mediate the crystallization and polymorphic direction process. Additionally, an overview is provided into the crystallographic structures of the four polymorphic forms of PABA and their similarities in terms of both their molecular structure and intermolecular packing. The potential for transferring the fundamental understanding of PABA crystallization to address larger-scale crystallization processes and their control through integrating crystal morphology control is also highlighted.

## Graphical abstract

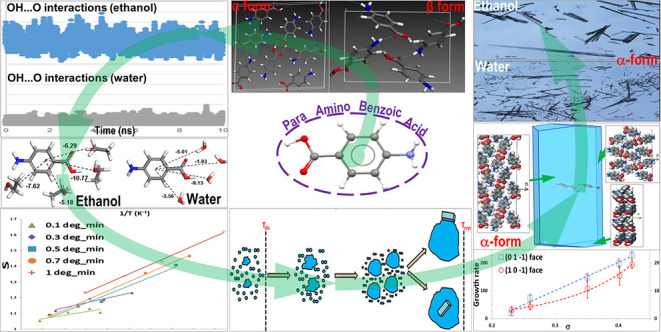

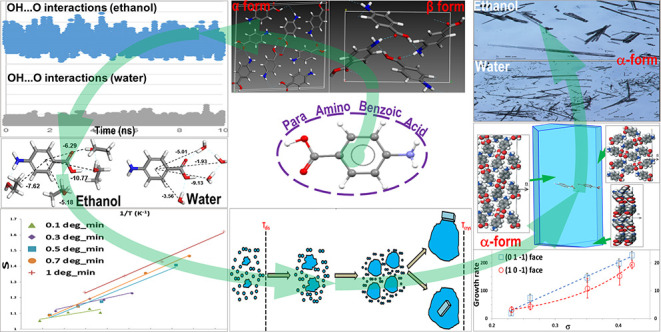

## 1 Introduction

A significant grand challenge for the physical-chemical sciences is understanding and controlling the transition pathway associated with the assembly of molecules from their supersaturated solvated state into crystalline solids, which plays a critical role in the design and operation of solution-phase crystallization processes.^1–3^ Such processes still present one of the most reliable and sustainable methods to isolate, purify, and separate crystalline materials and produce them with the required properties for their subsequent formulation and manufacture. The science underpinning the crystallization process, as summarized in Figure 1, encompasses the competitive actions of the nucleation (3D) and growth (2D) processes within the solution crystallization environment, notably the solute concentration, supersaturation, temperature, and solvent nature. Many organic crystalline materials display polymorphism with different forms having very different physicochemical properties such as bioavailability, stability, and formulatability. Thus, it can be crucial to produce crystalline ingredients in their correct (desired) polymorphic form as part of the development of an effective and sustainable crystallization process.

**Figure 1:**
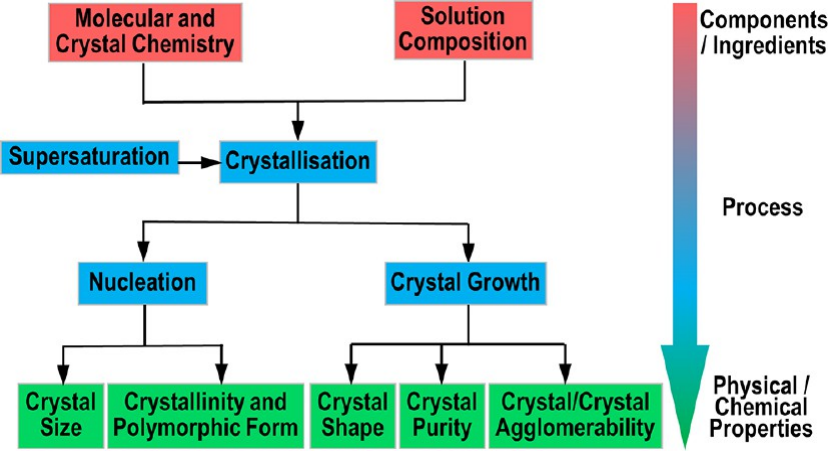
Schematic diagram showing the role played by the fundamental parameters of crystallization (nucleation and growth) in directing the physical properties of the resulting solid forms (data derived from^3^).

The formation and structural stability of the intermolecular solvated solute and/or solute cluster structures in solution, pre- and post-nucleation have been closely associated with the crystallization environment, e.g., solvent selection,^4–6^ temperature, and solute concentration^2,3,6–10^ . Such structures can, in turn, affect the crystallization behavior and, through this, result in the formation of different polymorphic forms of the material. Therefore, knowledge of the structural environment within the solution phase can form a key part of the science base which is needed for a fundamental understanding of the crystallization process design required for product development. The latter has the potential to impact on the product efficacy, stability, and time-to-market reduction.^2^

Molecular and crystallographic modeling using empirical force fields can be used to characterize intramolecular and intermolecular interactions within the crystal structures of organic crystalline materials in terms of understanding their molecular, solution-state, solid-state, and surface chemistry. Such predictions can also be used to examine the likely intermolecular chemistry involved in both solute solvation and solute cluster formation within the solution phase using, for e.g., molecular dynamics (MD) modeling^2,11,12^ along with complementary experimental characterization, such as X-ray diffraction (XRD), nuclear magnetic resonance (NMR), thermal analysis,^13^ and solution infrared (IR) spectroscopy.^12,14^ Such studies can also provide information on the physical-chemical properties of the solid forms, such as their polymorphic stability,^15,16^ morphology and surface chemistry,^17^ habit modification,^4,8,18^ surface wettability,^19^ and active pharmaceutical ingredient (API)/excipient compatibility.^20^

*p*-Aminobenzoic acid is a white crystalline substance with a molecular formula of (NH_2_)­(C_6_H_4_)­(COOH). This organic compound is also known as vitamin B10 and can also deliver good UV absorption and antifibrotic properties and was once widely used for manufacturing sunscreen (see^21^). In comparison to representative pharmaceutical materials,^16,22^ analysis of the molecular descriptors and crystallographic structural data (e.g., unit cell parameters (*a*, *b*, *c* and β), number of molecules in the asymmetric cell/unit cell (*Z*/*Z*′)) for PABA (Table 1) reveals that PABA has a relatively low molecular weight (137.14 g mol^–1^) with lower than average numbers of potential hydrogen-bond (H-bond or HB) donors (3) and acceptors (2) and aromatic rings (1), with an average number of rotatable bonds (3) in terms of molecular flexibility, when compared to the distribution of the approved pharmaceuticals.^16,22,23^ Nonetheless, the compound is consistent with that expected as a representative small-molecule pharmaceutical compound.

**Table 1:** Characteristic Molecular Descriptors and Crystallographic Structural Data for the *p*-Aminobenzoic Acid Polymorphs^a^

material descriptor	α-form	β-form	γ-form	δ-form
ref code	AMBNAC06 ^42^	AMBNAC04 ^41^	AMBNAC09 ^48^	AMBNAC14 ^47^
molecular weight (g mol^–1^ **)**	137.14	137.14	137.14	137.14
molecular volume (Å^3^)	117.32/118.34	124.86	119.52/121.48	121.98
molecular surface area (Å^2^)	138.40/138.30	142.94	139.63/141.62	139.09
melting point (°C)	187.3 ^44^	140.0 ^46^		
H-bond donors	2/2 (O_2_, N_1_/O_4_, N_2_)	2 (O_1_, N_1_)	2/2 (O1, N_1_/O_3_, N_2_)	2 (O_2_, N_1_)
H-bond acceptors	3/3 (O_1_, O_2_, N_1_/O_3_, O_4_, N_2_)	3 (O_1_, O_2_, N_1_)	3/3 (O_1_, O_2_, N_1_/O_3_, O_4_, N_2_)	3 (O_1_, O_2_, N_1_)
rotatable bonds	3/3	3	3/3	3
space group	*P*2_1_/*n* ^42^	*P*2_1_/*n* ^41^	*Pna*2_1_ ^48^	*Pn* ^47^
*Z*/*Z*′	8/2 ^42^	4/1 ^41^	8/2 ^48^	2/1 ^47^
*a* (Å)	18.571 ^42^	6.278 ^41^	26.9945 ^48^	6.4341 ^47^
*b* (Å)	3.843 ^42^	8.583 ^41^	3.7322 ^48^	4.6151 ^47^
*c* (Å)	18.632 ^42^	12.365 ^41^	12.6731 ^48^	10.5313 ^47^
β (deg)	93.67 ^42^	100.13 ^41^	90.0 ^48^	100.73 ^47^
cell volume (Å^3^)	1327.06 ^42^	655.91 ^41^	1276.80 ^48^	307.25 ^47^
packing coefficient	0.692	0.720	0.727	0.754
void space (%)	27.1	24.4	22.6	19.9
density (g cm^–3^)	1.37	1.39	1.43	1.48

a Note: The void space was calculated using CCDC’s Mercury with both the probe radius and the grid spacing being 0.2.^23^

The varied molecular and intermolecular chemistry of PABA gives rise to its crystallization and solid-form behavior, and as such, this compound forms a useful model compound for the fundamental studies, notably for examining the detailed molecular-scale structural pathway from its solvated molecular state through to its intermolecular solute clustering within the solution phase to its 3D nucleation of stable clusters within the supersaturated solution phase. Such clusters subsequently facet at the nanoscale and, through this, develop in time through cooperative 2D growth on all of the individual crystal facet surfaces, leading to the formation of a population of crystals at the microscale (Figure 2). Many of these various pathway steps can be mediated through the solvent selection process, impacting, in turn, on the physical-chemical properties of the resultant solid forms produced, notably their crystal morphology, surface properties, and polymorphic form.

**Figure 2:**
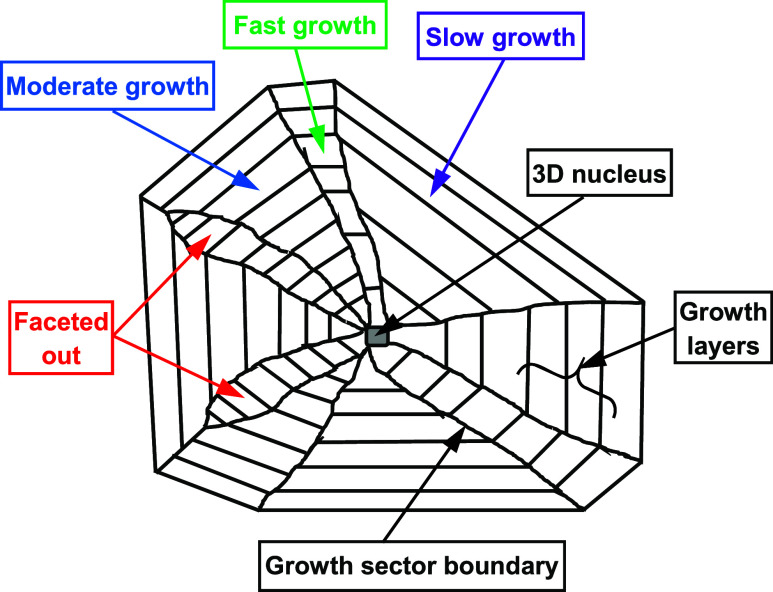
Schematic diagram of a hypothetical ‘cut’ through a crystal particle, showing the development of a fully faceted crystal from its initial 3D nucleation site (data derived from^3,24^ ).

Drawing upon the above perspective, this review paper seeks to overview recent research on the crystallization of PABA from the solution phase focusing on the areas of its molecular properties and crystallographic structures; crystal chemistry and intermolecular interactions; crystal morphology and surface chemistry; solution properties and metastability; solute clustering and nucleation; crystal growth; and polymorph selection.

This review encompasses 43 PABA-related research papers including 16 contributions from the Leeds group, with a focus on the formation of its α-polymorphic form and how the crystallization directs the formation of this form and how this process is mediated by solvent selection, molecular ordering, and processing conditions within the solution phase. In addition, and drawing down upon a recent review by Cruz-Cabeza et al.,^25^ the paper also provides a comparative overview of the crystal structures for all of the four known forms (α, β, γ, and δ) of PABA, highlighting, in particular, the structural similarities between these forms in terms of their molecular conformations, intermolecular interactions, and crystal chemistry.

The authors also wish to highlight that this review encompasses research carried out by the Leeds group over the past decade, which has involved many co-workers including contributions from researchers, notably Steve Caddick,^26^ Diana Corzo Camacho,^7,27,28^ Robert Hammond,^2,5,7,9,26,27,29^ Peter Kaskiewicz,^9,30–32^ Xiaojun Lai,^7,26,27,30,33^ Tariq Mahmud,^28^ Siti Mohd Noor,^28^ Roisín O’Connell,^34^ Jonathan Pickering,^5^ Ian Rosbottom,^2,5,9,27,29,34–36^ Mariana dos Santos,^7^ Dimitrios Toroz,^7,27,29^ Thomas Turner,^2,7,13,26,27,31,33,34,37^ Nicholas Warren,^9,30,31^ and Guanyi Xu.^30^

Details of the materials and experimental and modeling methods used to investigate the molecular and crystallographic properties, solution- and solid-phase characteristics, nucleation and growth, morphology, and surface chemistry of PABA have been detailed in the associated cited literature, e.g., (base-ref)^2,3,5,7,9,26,27,29,33,34,36,38^ with the overall interconnectivity of the work summarized in the workflow shown in Figure 3.

**Figure 3:**
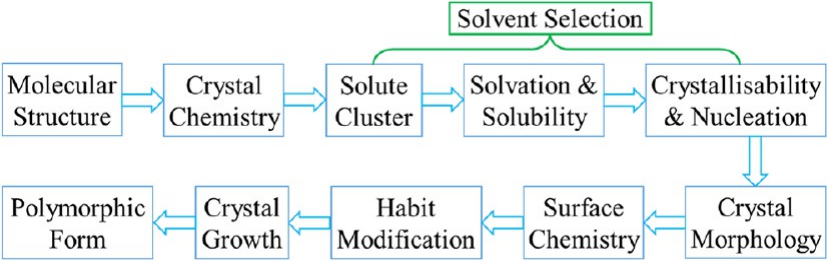
Workflow from the molecule and cluster to crystal and surface chemistry including solvent selection and solid-form properties.

## 2 Crystallographic Structures

PABA consists of three molecular moieties: an aromatic benzene ring substituted with a carboxylic acid and an amino side group, with these two being para with respect to each other (Figure 4).

**Figure 4:**
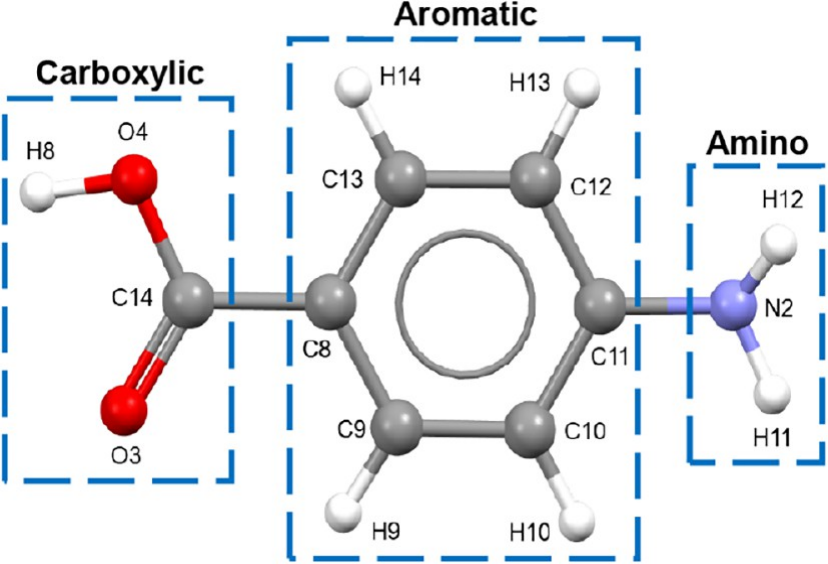
Molecular structure of *p*-aminobenzoic acid with the atom notations and the three molecular functional groups (moieties): carboxylic acid, aromatic ring, and amino group as indicated in the three boxes (data derived from^2^).

### 2.1 Crystallographic Structures and Their Unit Cells

The crystal structures of the α- and β-forms of PABA were first solved with CCDC’s cifcodes: AMBNAC01 ^39^ and AMBNAC,^40^ respectively, and their structures were later refined (e.g., AMBNAC04,^41^ AMBNAC06,^42^ AMBNAC10/11 ^43^). The α- and β-forms are known to have an enantiotropic relationship, with the α-form being found to be the more stable form at a higher temperature than the β-form. The close solubilities of these two forms led to the exact enantiotropic transition temperature being in some doubt with studies to date placing this between 13 and 25 °C.^26,44–46^ Overall, an analysis of the previous studies of PABA crystallization has highlighted that the preparation of the β-form can be both problematic and quite solvent-dependent.

More recently, further polymorphs have been discovered, notably the δ-form (AMBNAC14–18 ^47^) crystallized at high pressures and the γ-form (AMBNAC09 ^48^) obtained through crystallization in the presence of aqueous selenous acid.^48^ Recently, Cruz-Cabeza et al.^25^ have reviewed PABA’s polymorphic crystal chemistry, revealing the γ-form to be very similar to the α-form, while, in contrast, examination of the δ-form structure has revealed some similarities with both the α- and β-forms.

Reflecting its molecular structure encompassing both hydrogen donors and acceptors, PABA has been found to form a number of solvates and cocrystal solid forms under various crystallization conditions. For example, a solvate with dimethylformamide was crystallized using the antisolvent approach,^49^ while a nitromethane solvate was formed from the ethanol and nitromethane mixture.^34^ A number of cocrystals with ketoprofen,^50^ sulfamethazine,^51^ 6-methyluracil, barbituric acid, 2-hydroxybenzamide, and 4-hydroxybenzamide,^52^ and also one salt with emoxypine^52^ have been prepared by solvent evaporative crystallization. Mechanochemical methods with solvent assistance have also been used to crystallize cocrystals with ketoconazole,^53^ ciprofloxacin,^54^ and antipyrine and phenazine.^55^ New cocrystal forms with chlordiazepoxide^56^ has been obtained by dry-grinding approaches, while an agitated solution slurry method has been used to cocrystallize with acetazolamide.^57^

The characteristic molecular descriptors and associated crystallographic structural data for PABA (α, β, γ, and δ forms) are summarized in Table 1. The crystal structures of both the more commonly occurring α- and β-forms are centrosymmetric monoclinic with a *P*2_1_/*n* space group, while the δ- and γ-forms have polar structures with *Pn* and *Pna*2_1_ space groups, respectively. Examination of the crystal chemistry of the α- and γ-forms reveals that both comprise dimeric carboxylic packing motifs (Figure 5a,c), with each having two crystallographically independent dimer units within the asymmetric unit, leading to a total of eight molecules in the unit cell. In contrast, the β-form has a single molecule (Figure 5b) within a tetramolecular asymmetric unit in the unit cell, and the δ-form has a single bimolecular molecule (Figure 5d) asymmetric unit. Interestingly, the δ-form has been found to be the most closely packed polymorph with the highest density (1.48 g cc^–1^) and the lowest void space (19.9%) among the four known forms in an order of δ, γ, β, and α forms (Table 1, rows 16–18). This might suggest the δ-form to be the stable polymorphic form, but this postulation is yet to be validated.

**Figure 5:**
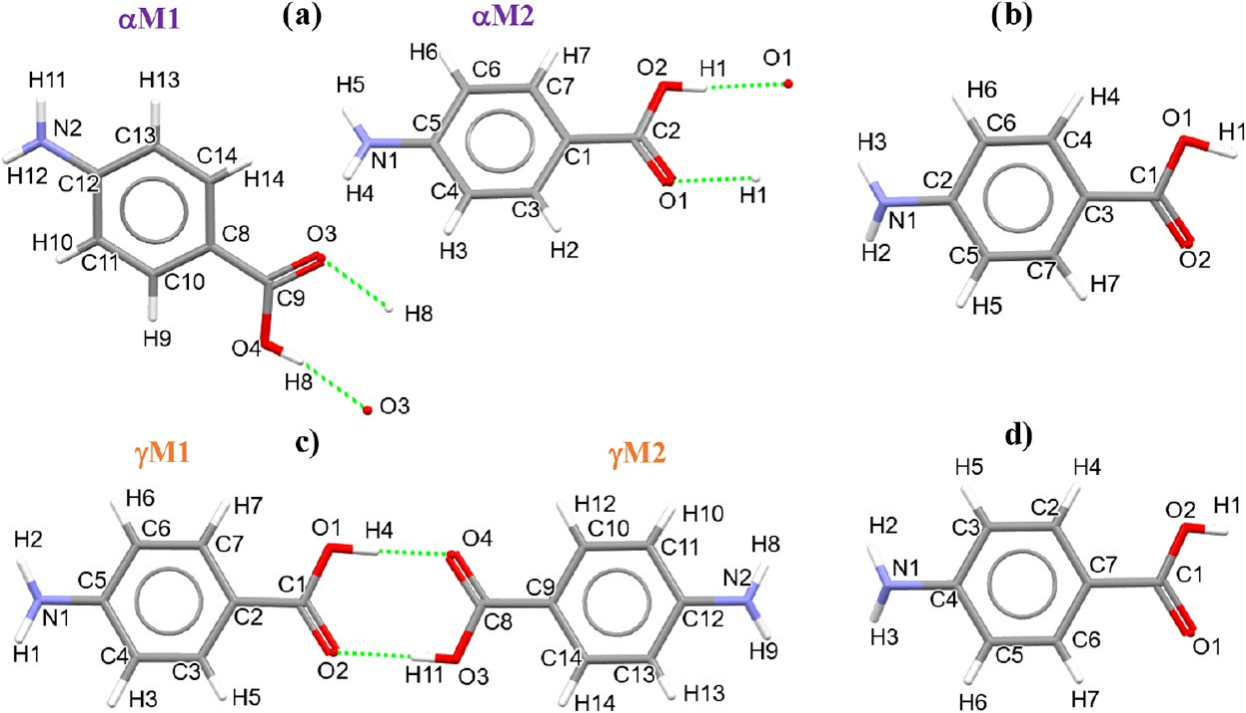
Molecular structures of *p*-aminobenzoic acid: (a) α-form (AMBNAC06) with two molecules (αM1, αM2) in the asymmetric unit (two hydrogen-bonding dimers (Table 2)); (b) β-form (AMBNAC04) with one molecule in the asymmetric unit; (c) γ-form (AMBNAC14) with two molecules (γM1, γM2) in the asymmetric unit (one hydrogen-bonding dimer (Table 2)), and (d) δ-form (AMBNAC09) with one molecule in the asymmetric unit.

### 2.2 Comparison between Molecular Conformations and Intermolecular Packing for the Four Forms

Examination of the crystal packing networks reveals that π–π stacking interactions always occur along their *b* axis, with the stronger interactions being expected to be the γ- and α-forms followed by the β- and δ-forms, based on their π–π stacking configurations (Table 2).

**Table 2:** Molecular and Crystal Packing Details of Four PABA Forms (α, β, δ, and γ)

form	α	β		δ	γ
ring normal distance (Å)	3.397		3.427	3.299	3.306
ring center-to-center distance (Å)	3.843		4.332	4.615	3.732
offset (Å)	1.800		2.650	3.230	1.730
stacking type	H2H		H2T	H2H	H2H
stacking angle (deg)	141.17	67.79		91.25	135.63
dimer	Y	N		N	Y
number of dimers	2 (αM1-αM1 & αM2-αM2)	N/A		N/A	1 (γM1-γM2)
HB distance (Å)	O_4_H_8_···O_3_: 1.813	O_1_H_1_···N_1_: 1.730		O_2_H_1_···N_1_: 1.920	O_1_H_4_···O_4_: 1.631
	O_4_H_8_···O_3_: 1.813	N_1_H_2_···O_2_: 2.186		N_1_H_2_···O_1_: 2.267	O_3_H_11_···O_2_: 1.756
	N_2_H_11_···O_1_: 2.131	O_1_H_1_···N_1_: 1.730		O_2_H_1_···N_1_: 1.920	N_1_H_1_···O_4_: 2.010
	N_2_H_11_···O_1_: 2.131	N_1_H_2_···O_2_: 2.186		N_1_H_2_···O_1_: 2.267	N_2_H_8_···O_2_: 2.597 (not a HB)
HB angle (deg)	O_4_H_8_···O_3_: 166.05	O_1_H_1_···N_1_: 159.95		O_2_H_1_···N_1_: 164.57	O_1_H_4_···O_4_: 174.58
	O_4_H_8_···O_3_: 166.05	N_1_H_2_···O_2_: 163.98		N_1_H_2_···O_1_: 160.80	O_3_H_11_···O_2_: 166.10
	N_2_H_11_···O_1_: 164.65	O_1_H_1_···N_1_: 159.95		O_2_H_1_···N_1_: 164.57	N_1_H_1_···O_4_: 171.66
	N_2_H_11_···O_1_: 164.65	N_1_H_2_···O_2_: 163.98		N_1_H_2_···O_1_: 160.80	N_2_H_8_···O_2_: 142.97 (not a HB)
torsion angle (deg)	O_4_H_8_···O_3_C_9_: 131.75	O_1_H_1_···N_1_C_2_: 109.25		O_2_H_1_···N_1_C_4_: 123.92	O_1_H_4_···O_4_C_8_: 99.94
	O_4_H_8_···O_3_C_9_: −131.75	N_1_H_2_···O_2_C_1_: 110.08		N_1_H_2_···O_1_C_1_: −128.79	O_3_H_11_···O_2_C_1_: 101.34
	N_2_H_11_···O_1_C_2_: 160.11	O_1_H_1_···N_1_C_2_: −109.25		O_2_H_1_···N_1_C_4_: −123.92	N_1_H_1_···O_4_C_8_: −88.55
	N_2_H_11_···O_1_C_2_: −160.11	N_1_H_2_···O_2_C_1_: −110.08		N_1_H_2_···O_1_C_1_: 128.79	N_2_H_8_···O_2_C_1_: 146.08 (not a HB)
ring center-to-center distance of 2 molecules in a dimer (Å)	αM1-αM1:0.421	N/A		N/A	N/A
	αM2-αM2:0.450				
ring plane angle of 2 molecules in a dimer (deg)	αM1-αM1:0	N/A		N/A	γM1-γM2:1.13
	αM2-αM2:0				
molecule *L* × *W* (Å)	about 8 × 4				

Table 3 presents the root mean-squared deviation (RMSD) and also the maximum deviation between the six molecular structures (Figure 5): two for the α-form (αM1, αM2), one for the β-form, two for the γ-form (γM1, γM2), and one for the δ-form in their asymmetric units. Their overall RMSDs (Table 3, third row) were found to be in the range of 0.0157–0.0972 Å with their maximum deviations being 0.0381–0.1851 Å, indicating that the six molecular structures for all four PABA forms are quite similar to each other. As a result, the three H-bond dimers of the α-form (αM1-αM1, αM2-αM2) and γ-form (γM1-γM2) were also found to be very similar (Figure 6), though the two H-bond lengths of the γ-form dimer were found to be shorter than those for the α-form and also not equal (Figure 8b and Table 2).

**Figure 6:**
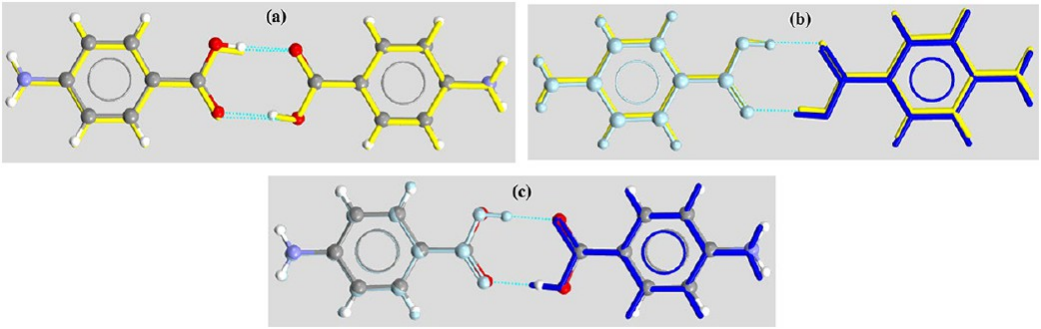
Comparisons between 3 dimers from α- and γ-forms: (a) α-form dimer1 (αM1-αM1) (yellow) + α-form dimer2 (αM2-αM2) (ball&stick); (b) α-form dimer1 (αM1-αM1) (yellow) + γ-form dimer (γM1-γM2) (dark blue stick + light blue ball&stick); and (c) α-form dimer2 (αM2-αM2) (ball&stick) + γ-form dimer (γM1-γM2) (dark blue stick + light blue ball&stick).

**Table 3:** Similarity Comparisons of All Molecules Including 2 from the α-Form, 1 from the β-Form, 2 from the γ-Form, and 1 from the δ-Form

molecule #1	αM1	αM1	αM2	αM1	αM2	αM1	αM2	αM1	αM2	β	β	β	δ	δ	γM1
molecule #2	αM2	β	β	δ	δ	γM1	γM2	γM2	γM1	δ	γM1	γM2	γM1	γM2	γM2
RMSD (Å)	0.0591	0.0972	0.0864	0.0576	0.0565	0.0220	0.0157	0.0703	0.0627	0.0462	0.0465	0.0497	0.0828	0.0875	0.0729
max. deviation (Å)	0.1136	0.2014	0.1771	0.1096	0.1192	0.0381	0.0239	0.1373	0.1244	0.0932	0.0784	0.1259	0.1700	0.1851	0.1481

Analysis of the H-bonds, as shown in Table 4, reveals that they are stronger in the β-form structure than in the α-form, as evidenced by the OH···O and NH···O bond lengths in the β-form being ca. 5 and 7%, respectively, shorter than those in the α-form.

**Table 4:** H-Bond Geometrical Details of the α-, β-, γ-, and δ-Form PABA Molecules with the Contribution Donor (D) and Acceptor (A) Sites Together with Their Respective Polarizability

polymorph	H-bond	H···A (Å)	D···A (Å)	D-H (Å)	D–H···A (deg)
α-form (1st molecule)	O2H1···O1	1.836	2.650	0.819	172.23
	N1H4···O3	2.574	3.368	0.860	154.05
α-form (2nd molecule)	O4H8···O3	1.813	2.616	0.820	166.05
	N2H11···O1	2.131	2.969	0.860	164.65
β-form	O1H1···N1	1.730	2.754	1.065	159.95
	N1H2···O2	2.186	3.045	0.884	163.98
γ-form (1st molecule)	O1H4···O4	1.631	2.610	0.982	174.53
	N1H1···O4	2.010	2.958	0.955	171.66
γ-form (2nd molecule)	O3H11···O2	1.756	2.631	0.893	166.10
	N2H8···O2	2.597	3.305	0.838	142.97
δ-form	N1H2···O1	2.267	3.127	0.900	160.80
	O2H1···N1	1.920	2.720	0.821	164.57

### 2.3 Comparison between Crystal Packing for the Four Forms

A more detailed comparison between the solid-state chemistry of the more commonly observed α- and β-forms^35^ reveals that the β-form (low temperature) has a characteristic 4-membered H-bonding ring comprising identical pairs of alternating OH···N and NH···O interactions^43^ (Figure 7b) with weak head-to-tail (H2T) π–π aromatic stacking in the *b* axis. The β-form has been observed to be quite difficult to crystallize^46,58^ even at temperatures below its charaterized transition temperature, notably being only reliably formed from an aqueous slurry of the α-form through conversion at ca. 5 °C over a ca. two-week period.^37^ In contrast, the α-form has strong H-bonding carboxylic acid OH···O dimer interactions coupled with strong head-to-head (H2H) π–π stacking and NH···O interactions (Figure 7a). The same type of H-bonding and H2H π–π infinite stacking configurations was found in the crystal structures of α- and γ-forms. In contrast, both the β- and δ-form structures present the H2T H-bonding, but the stronger π–π stacking in the β-form is related by inversion whereas in the δ-form it is related by translation.^25^

**Figure 7:**
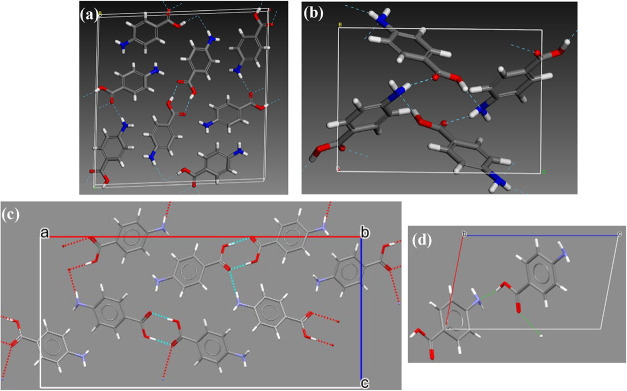
Crystal packing structures of *p*-aminobenzoic acid: (a) α-form; (b) β-form; (c) γ-form; and (d) δ-form with their H-bonding networks (data derived from^2,35^ ).

### 2.4 Comparison between the α and γ (*Z′* = 2) Structures

As shown in Table 1, both the α- and γ-forms have two molecules within the asymmetric units (*Z′* = 2), referring to αM1 and αM2 for molecules 1 and 2 of the α-form and γM1 and γM2 for molecules 1 and 2 of the γ-form (Figure 5a,c). Examination of the H-bond networks for the α-form (Figure 8a and Table 2) reveals that two H-bonding dimers (αM1-αM1 and αM2-αM2) were formed via two carboxylic (COOH) H-bonds (Figure 8a, highlighted by purple and blue dashed ovals), with each being formed by the individual molecules (αM1 or αM2) themselves and having the same bond length of 1.836 Å. The two centrosymmetric dimers (Figure 8a) form a packing pattern in the a–c plane with an angle of ∼36° between them, and these two dimers are not equivalent due to the molecular structures of αM1 and αM2 not being identical. The α-form has distinct differences in H-bonding between the two dimers (αM1-αM1 and αM2-αM2), as shown in Table 2 and Figure 8a, with dimer one (αM1-αM1) being found to have two H-bonds (Table 5) between its carboxyl head groups and the amino tail groups of dimer two (αM2-αM2) with their bond lengths being equal (2.131 Å). Interestingly, the amino tail groups of dimer one (αM1-αM1) were found not to produce any H-bonding interactions with the carboxyl head groups of dimer two (αM2-αM2) due to the long distances between their amino (αM1) and carboxyl (αM2) groups (Figure 8a, red-colored distances of 2.574 Å). Therefore, for dimer two (αM2-αM2), two H-bonds were formed between its own amino tail groups and the carboxyl head groups of dimer one, but without H-bonding interactions between its carboxyl head groups and the amino tail groups of dimer one (Table 5).

**Figure 8:**
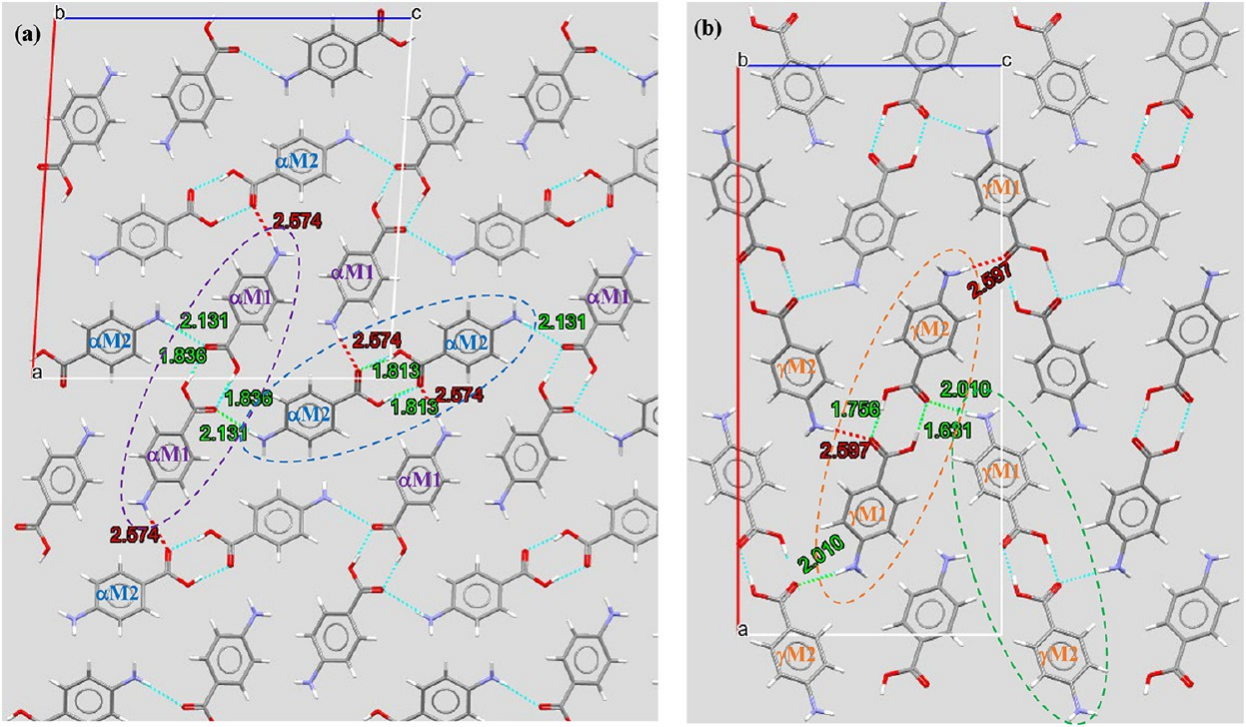
Crystal packing structures: (a) α form with 2 dimers (αM1-αM1: purple dashed oval and αM2-αM2: blue dashed oval), H-bond distances (green), and non-H-bond distances (red) and (b) γ form with one dimer (γM1-γM2) but two packing orientations (brown and green dashed ovals), H-bond distances (green), and non-H-bond distances (red).

**Table 5:** H-Bonding Interactions (Amino HB Donor and Carboxylic HB Acceptor) and Their Numbers for α- and γ-Forms

	M1		M2		M1-M1		M2-M2		M1-M2		(M1-M1)–(M2-M2)		(M1-M2)–(M1-M2)	
PABA form	HB donor	HB acceptor	HB donor	HB acceptor	HB donor	HB acceptor	HB donor	HB acceptor	HB donor	HB acceptor	HB donor	HB acceptor	HB donor	HB acceptor
**α**	N (0)	Y (1)	Y (1)	N (0)	N (0)	Y (2)	Y (2)	N (0)			Y (2)	Y (2)		
**γ**	Y (1)	N (0)	N (0)	Y (1)					Y (1)	Y (1)			Y (1)	Y (1)

In the γ-form, only one H-bond dimer (γM1-γM2) (Figure 8b, highlighted by the brown dashed oval) was observed through two COOH H-bonds between the two molecules (γM1 and γM2) with shorter and nonequal H-bond lengths of 1.631 and 1.756 Å when compared to the equal length for the two dimers of the α-form. Both γM1 and γM2 have one H-bond acceptor (C=O) and one H-bond donor (amino; Figure 8b and Table 5). Furthermore, a single H-bond between the dimers of the γ-form with different packing orientations (Figure 8b, brown and green dashed ovals) was formed between the carboxyl group of γM2 (brown dashed oval) and the amino group of γM1 (green dashed oval) with a H-bond length of 2.010 Å, while no H-bond was formed between the carboxyl group of γM1 and the amino group of γM2 due to the long O–H distance of 2.597 Å, which might be caused by the shorter H-bond length of 1.631 Å in the dimer to pull the amino group of γM2 away from the carboxyl group of γM1. The two dimers (Figure 8b) in different orientations form a packing pattern in the a–c plane with a smaller angle of ∼16° between them when compared to 36° for the α-form.

Further examinations reveal that the aromatic rings of the two molecules within the two dimers (αM1-αM1 and αM2-αM2) for the α-form are closely parallel to each other (Table 2, column 2), while the rings in the γ-form have an angle of 1.13° between the two molecules in the γM1-γM2 dimer (Table 2, column 5), which might be due to the stronger π–π molecular stacking interactions for the γ-form. Furthermore, the potential second amino–oxygen H-bond in the γ-form, as shown in red (Figure 8b and Table 2), has a much longer distance (2.597 Å) between H and O compared with the bond distances of 1.631–2.131 Å for the H-bonds in both the α- and γ-forms. Similarly, the potential two amino–oxygen H-bonds in the α-form, as shown in red (Figure 8a), also have a long distance (2.574 Å) between H and O.

Overall, neither of these two structures are optimal in terms of their H-bond saturation, suggesting the potential for the existence of a further polymorphic form that is completely saturated in terms of H-bonding availability. This though might require changes to the molecular conformation to enable the H-bond formation, which may or may not be physically realistic.

## 3 Molecular, Crystal, and Surface Chemistry

### 3.1 Molecular Conformational Stability

Figure 9 shows results from an analysis of the relative conformational stabilities^29^ of both α- and β-form molecules with respect to their carbonyl (a) and amino (b) groups using density functional theory (DFT). This reveals the β-form conformation to be slightly distorted, with respect to the more stable α-form. In the α-form conformer, the carboxyl acid group was found to be very close to the planar conformational minima (0°, ca. 0 kJ mol^–1^ of relative energy), with the amino group exhibiting a slight pyramidal bend located at 12.24° with a relative energy of 1.3 kJ mol^–1^, close to the pseudopyramidal conformational minima (23.8°, 0.003 kJ mol^–1^ of relative energy). For the β-form molecule, the carboxyl acid group (ca. 10°, ca. 1.0 kJ mol^–1^ of relative energy) was found to be slightly rotated away from a planar conformation, while the amino group was found at 31.66° with a relative energy of 3.07 kJ mol^–1^, which is more pyramidal. This conformational distortion reflects the need for the molecules in the β-form strucutre to complete its tetramolecular H-bonded ring structure. As the α-form PABA conformation is closer to the minimum energy conformation (Figure 9), this difference would be expected to present a lower conformational energy barrier to the crystallization of the α-form when compared to the β-form, consistent with the known challenges associated with crystallizing the β-form.

**Figure 9:**
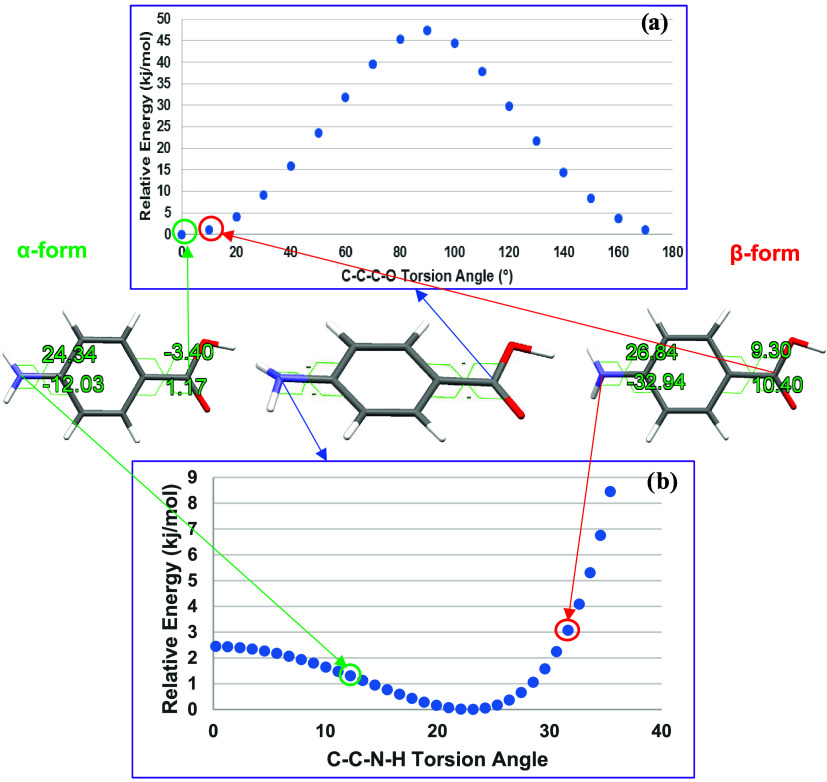
Conformational DFT analysis of α- and β-form PABA molecular structures using (a) rigid carboxyl acid (COOH) rotation and (b) pyramidal bend of amino (NH_2_) groups (data derived from^29^).

The examination of the α-form crystal structure revealed its characteristic rigid and almost planar COOH dimer structure (Figure 7a). This, together with the relative planarity of the related NH···O H-bond, results in the α-form’s tetramolecular core building block also remaining fairly planar. In contrast, the alternating OH···N and NH···O H-bonds characteristic of the β-form tend to draw the position of the N atoms toward the OH group and the H atom toward the C=O group, respectively (Figure 7b), leading overall to a more slightly distorted conformation for both functional groups within the core 4-membered H-bond ring in the β-form structure. Hence, it might be expected that while the strength of this core 4-membered H-bonding ring structure might provide a strong and stable arrangement for its subsequent development and growth (Table 4), its higher conformational deformation energy associated with solute cluster assembly during nucleation could be expected to lower its overall crystallizability.

### 3.2 Intermolecular Interactions and Lattice Energies

Calculation of the lattice energy for the α- and β-form structures together with their cumulative and discretized interaction energy convergence profiles, as shown in Figure 10, reveals that the α-form achieves more cohesive energy from its near intermolecular neighbors than the β-form.^35^ This indicates that the β-form needs the assembly of a larger intermolecular cluster of solute molecules at nucleation in order to form a stable lattice structure, when compared to the α-form. In turn, larger intermolecular cluster sizes imply lower nucleation rates and hence much longer induction times for the β-form and vice versa for the α-form. Further analysis of the lattice energy convergence data supports this hypothesis revealing that the α-form exhibits a sharp increase in the lattice energy from its closest interactions, while the β-form gains the lattice energy from intermolecular interactions at greater distances. Hence, it might be expected that higher supersaturations would lead to the formation of the α-form, while lower supersaturations would be expected to produce the β-form. Overall, this and the conformational analysis are consistent with the longer duration times needed at low temperatures for the enantiotropic transformation and recrystallization of the β-form from the α-form.^58^

**Figure 10:**
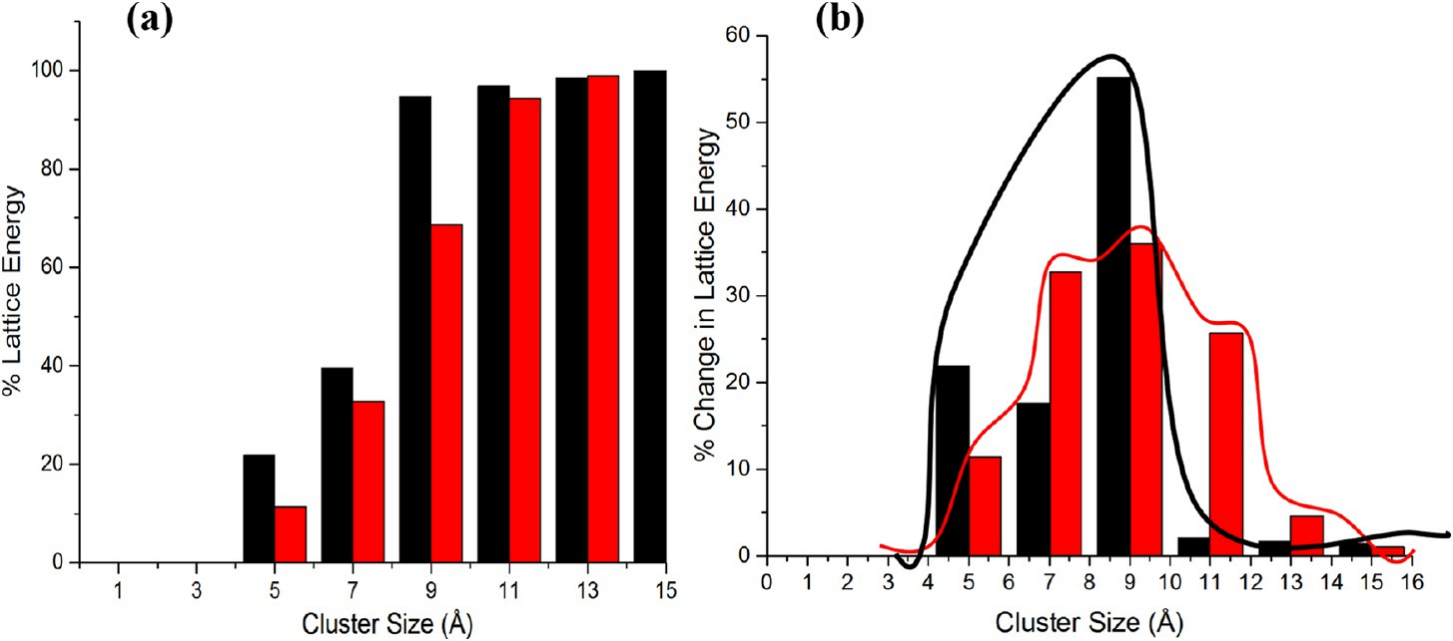
(a) Cumulative and (b) discretized lattice energy distributions of PABA as a function of the cluster size (black: α-form; red: β-form) (data derived from^2,35^ ).

Characterization of the bulk (intrinsic)^59^ intermolecular synthons^36^ reveals the strongest synthons to be Aα, Bα, Cα, and Dα for the α-form and Aβ, Bβ, Cβ, Dβ, and Eβ for the β-form polymorphs of PABA whose intermolecular configurations are given in Figure 11. For the α-form, these reveal the OH···O hydrogen-bonding dimers (synthon Aα) and π–π H2H infinite stacking interactions (synthon Bα) to be the most important intermolecular interactions in the α-form structure, followed by, in decreasing strength, the NH···O hydrogen-bonding dimers (synthon Cα) and dispersive π–π H2H vdW interactions (synthon Dα).^2,36^ These top four synthons in the α-form structure contribute ca. 72% to the total lattice energy of the α-form.

**Figure 11:**
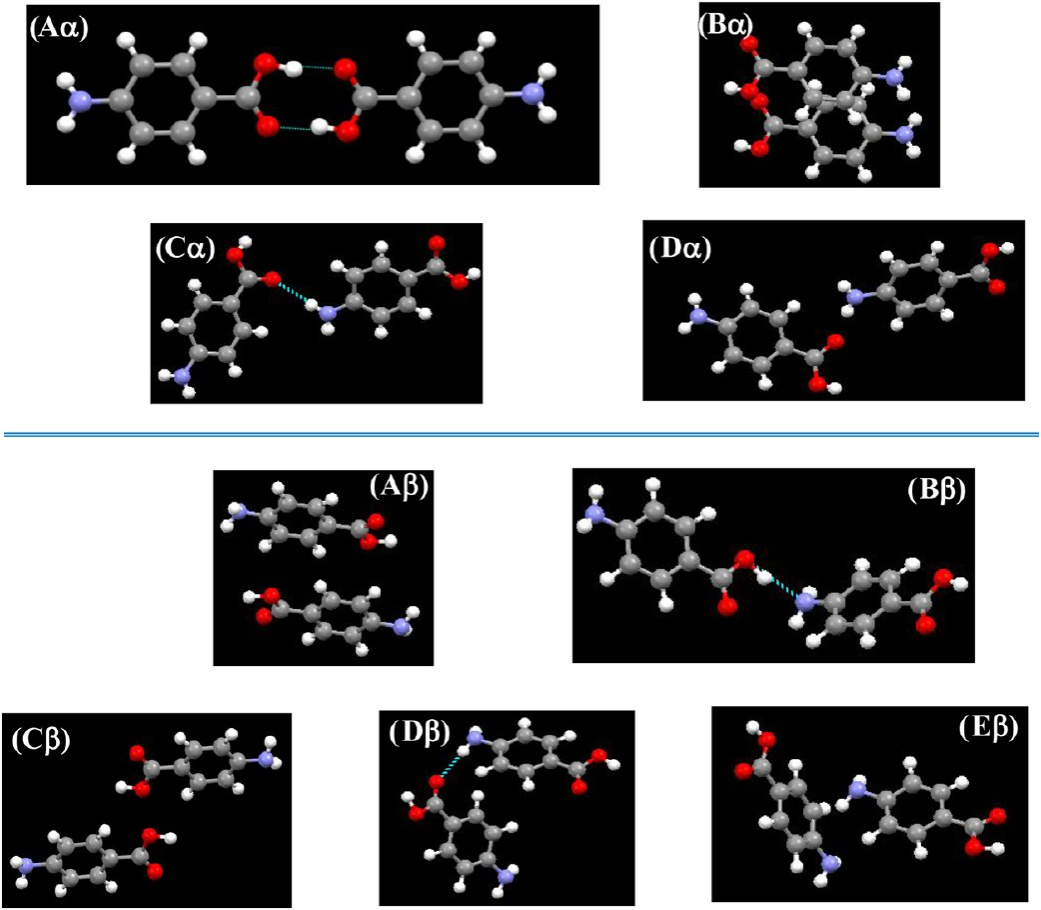
Top-strength-ranked intrinsic synthons for α-form (Aα, Bα, Cα, Dα) and β-form (Aβ, Bβ, Cβ, Dβ, Eβ) PABA crystals. The synthon strengths were calculated using the empirical interatomic potential (data derived from^2,27,36^ ).

In contrast, for the β-form, the strongest intermolecular interactions were found to be the H2T π–π dimer pair synthons (Aβ), followed by the OH···N H-bonding interactions (Bβ), displaced H2T vdW interactions (Cβ), NH···O H-bonding interactions (Dβ), and dispersive vdW interactions (Eβ). The top H2T π–π interactions (Aβ), together with the alternating NH···O (Bβ) and OH···N (Dβ) hydrogen-bonding interactions involved in the tetramolecular ring structure, were found to be the important synthons in β-form crystals.^2,36^ These top five synthons in the β structure make up a contribution of ca. 75% to the total lattice energy of the β-form.

As shown in Table 6, the different intermolecular interactions and packing of the α and β polymorphs lead to a higher lattice energy for the α-form together with quite different relative contributions of the material’s functional groups to the lattice energy, with the analysis indicating that the formation of the α-form might be expected to dominate during crystallization due to the energetic importance of its strong carboxylic dimer (Aα) intermolecular interaction, which alone contributes almost ca. 50% to the lattice energy. This compares to the β-form where the dispersive π–π H2T interactions and OH···N H-bonding interactions with the amino group are relatively more important in terms of this form’s thermodynamic stability.^36^

**Table 6:** Lattice Energy Together with the Percentage of Contributions from Three Functional Groups (Amino, Aromatic Ring, and Carboxyl Acid)^a^

	α-form	β-form
lattice energy (kcal mol^–1^)	–24.45	–22.73
NH_2_ (%)	15.3 (donor only)	23.8 (both donor and acceptor)
C_6_H_6_ (%)	39.8	42.5
COOH (%)	44.7	33.7

a The data are derived from ref 36.

Analysis of the individual intermolecular interactions (intrinsic synthons), as listed in Table 7, shows that the strongest interactions in the α-form were found to be the H-bonding dimers between the carboxylic acid groups, contributing approximately 23% of the calculated lattice energy. Interestingly, synthon Bα, which involves the more isotropic vdW forces due to π–π interactions between close-packed molecules stacking along the *b* axis, was found to contribute approximately 22% of the total calculated lattice energy.^2,36^

**Table 7:** Strongest Intermolecular Interactions from the Top Five Synthons for the α-form Crystals^a^

bond	intermolecular energy (kcal mol^–1^)	percentage contribution to lattice energy	dominating interatomic interaction type	COOH % contribution to interaction	C_6_H_4_ % contribution to interaction	NH_2_ % contribution to interaction	{101}	{101̅}	{011̅}
Aα	–5.7	23.1	OH···O H-bond	96.4	4.0	–0.4	0	1	1
Bα	–5.4	21.8	π–π stacking	14.5	72.6	13.0	0	1	1
Cα	–2.3	9.3	NH···O H-bond	41.7	20.7	37.6	0	0	2
Dα	–2.0	8.2	displaced H2H vdW	38.8	26.1	35.1	0	2	0
Eα	–2.3	9.2	vdW	79.9	21.0	–0.9	0	0	0
total	–18.7	71.6							

a Adapted from refs 2 and 36.

The functional group contribution analysis with respect to the lattice energy is further expanded in columns 5–7 (Table 7) by considering the difference in their % contributions to the intermolecular interaction strengths, both within the ranked lists for the α-form. For example, the carboxylic H-bonded dimers (Aα) were found to have over 96% of their interaction centered on the COOH group, while the π–π stacking interaction (Bα) was found to be more centered on the phenyl ring, with over 72% of the interaction contributed by the phenyl ring.^2,36^

Overall, the strongest synthons for the α-form crystal structure were found to be dominated by contributions from the carboxyl acid and phenyl ring groups in quite an anisotropic crystal chemistry compared to the β-form^2,36^ where the interactions were found to be more evenly distributed in 3D, yielding, in turn, a much more isotropic lattice structure for the β-form compared to the α-form.

These differences between the crystal chemistry of the α- and β-forms have been shown to directly correlate with the resulting physical properties of the crystals as highlighted by their measured axial thermal expansion coefficients, as shown in Figure 12, using variable-temperature powder X-ray diffraction.^33^ Generally, the thermal expansion coefficients of the *b* axis were found to be about 10 and 2 times larger than those of the *a* and *c* axes for α- and β-forms, respectively.^33^ The largest thermal expansion was found to be along the *b* axis for the α-form crystals, with its thermal expansion coefficient being 94.5 × 10^–6^ K^–1^ compared to 8.36 × 10^–6^ and 9.91 × 10^–6^ K^–1^ for the *a* and *c* axes where hydrogen bonding is made prevalent.^33^ This would be expected due to the much weaker and isotropic nature of the H2H π–π stacking interactions (Bα) (Figure 11) that exist along the *b* axis of the α-form crystal structure, while the *a* and *c* axes have H-bonds in plane with the strong COOH hydrogen-bonded dimer interactions (Aα) and NH···O bonds (Dα) (Figure 11) running ∼45° to the plane normal down the *a* and *c* axis directions.

**Figure 12:**
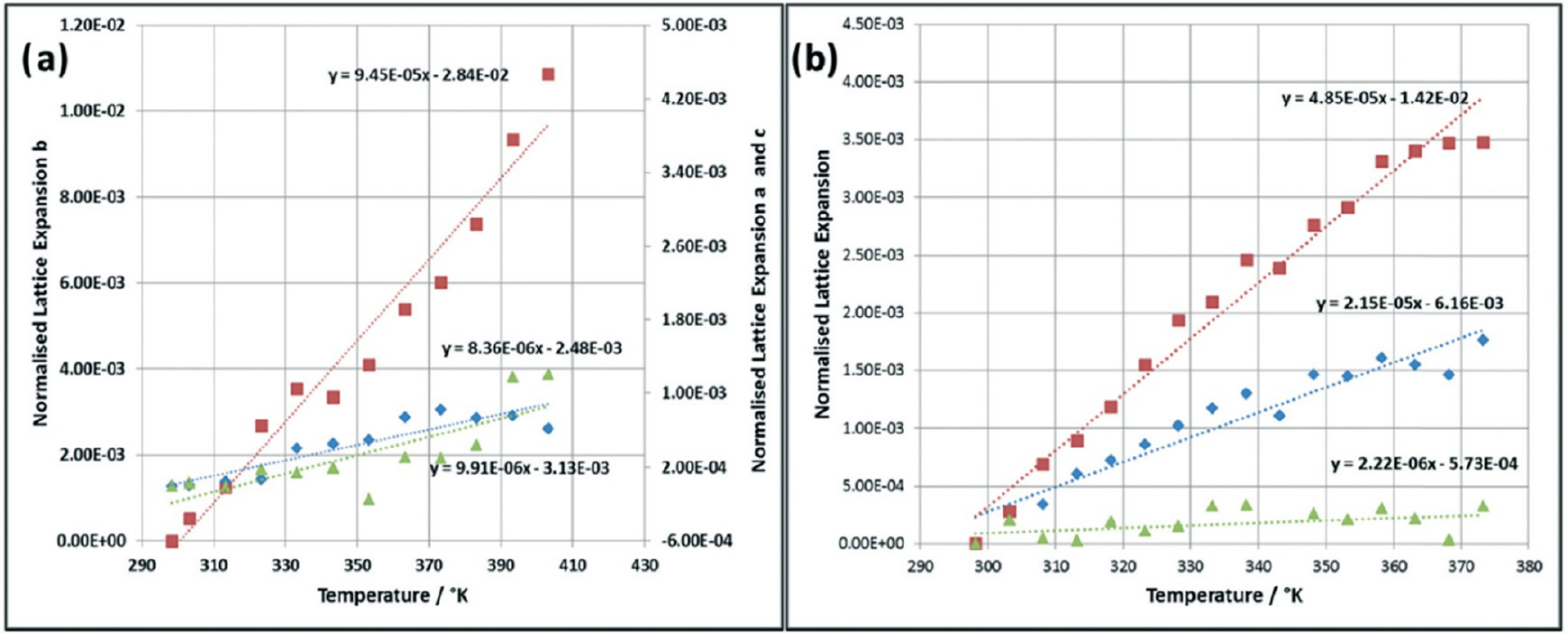
Normalized linear thermal expansion in the various crystallographic axes as a function of temperature for the (a) α polymorph and (b) β polymorph (blue diamonds = *a* axis, red squares = *b* axis, green triangles = *c* axis) (data derived from^33^).

### 3.3 Crystal Morphology and Surface Chemistry

Morphological predictions based on the crystallographic structures, shown in Figure 13,^36^ reveal that α-PABA would be expected to have a flat, lathe-like crystal morphology. The largest {101} faces were found to be predicted to have large surface areas consistent with weak vdW forces dominating the {101} surface’s π–π stacking interactions (Figure 13a) from the lower-energy synthons (J, M, and O) without any contribution from stronger top synthons involved (Table 7, column 8), corresponding to the much lower attachment energy and much higher saturation of the surface molecule (*ξ*), as shown in Table 8. For the side {10–1} face, three synthons (A, B, and D) made considerable contributions to the lattice energy (Table 7, column 9), and the surface interactions were found to contain H-bonding interactions (Figure 13b) and were hydrophilic in nature, and the capping {01–1} faces consist of π–π stacking interactions that are hydrophobic and dominated by the carboxylic acid dimer interactions (Figure 13c), as shown in Table 7 (column 10), for the contributions from strong synthons (A, B, C), reflecting the higher attachment energy and lower saturation of the surface molecule on the {01–1} habit faces (Table 8). These much weaker interactions for the {101} surface would be expected to be consistent with the much slower growth rate.

**Figure 13:**
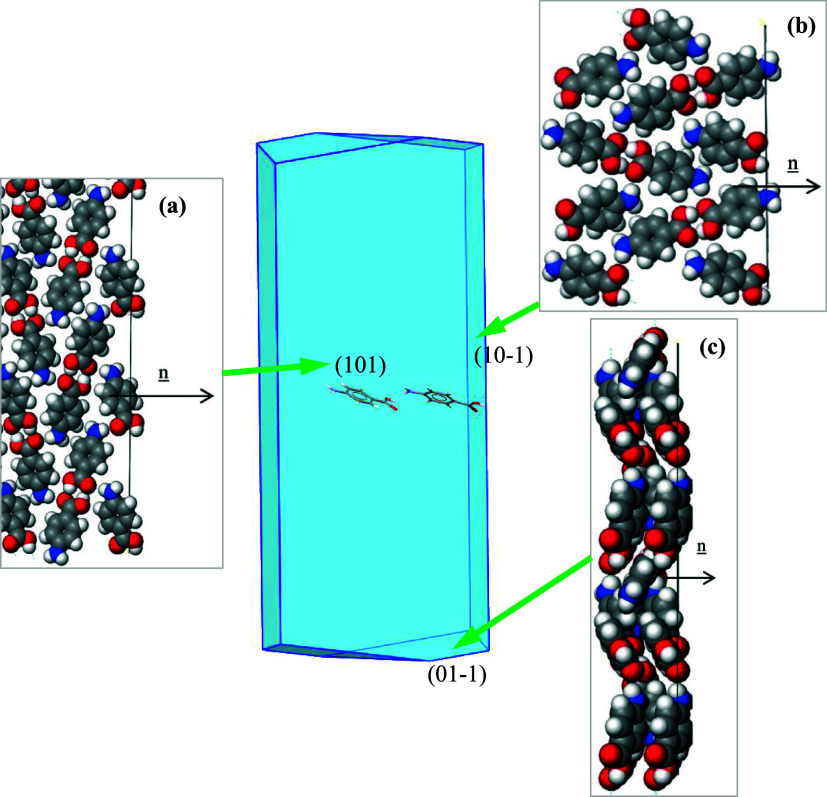
Crystal chemistry of the (a) (101), (b) (10–1), and (c) (01–1) surfaces of α-PABA with a space fill model of the side view (data derived from^36^).

**Table 8:** Slice, Attachment, and Anisotropy Factor (*ξ*) of the Important Faces Predicted by the BFDH Method of α-PABA^a^

face *{hkl}*	slice energy (kcal mol^–1^)	attachment energy (kcal mol^–1^)	*ξ* (%)
101	–24.5	–1.7	93.6
101̅	–14.1	–10.4	66.2
011̅	–9.2	–15.4	35.9

a The data are derived from ref 36.

## 4 Solution State, Solute Clustering, and Nucleation

Studies of the solution-phase nucleation behavior of the polymorphic forms of PABA have been previously reported (e.g.,^5,7,26,28,30,31,46,60–63^ ), using both molecular modeling and experimental measurements. Nappo et al.^63^ demonstrated a nonmonotonic dependence of the primary nucleation rate on the shear rate, while studies based on Ostwald’s rule demonstrated the important contribution of the growth process in terms of the polymorphic outcome, revealing also that molecular-scale mechanisms tend to dominate the nucleation process.^62,64^ Regarding nucleation mechanisms, it has been found that the intermolecular cluster growth via aromatic stacking potentially might be important,^60^ which is affected, in turn, by solution chemistry and hydrogen bonding. Sullivan et al.^58^ found solute dimerization and desolvation to be important rate-determining processes within the overall nucleation pathway. The effect of ultrasound on nucleation has also been investigated, revealing that ultrasound can disturb the assembly of the carboxylic acid dimers (synthon Aα) in the solution phase and, through this, can favor the crystallization of the β-form.^65^

### 4.1 Solute Solubility and Solution Ideality

Examination of the van’t Hoff plots (Figure 14) of the solubility data for ethanol (EtOH), acetonitrile (AcN), and water solvents^2,7^ from solution dissolution measurements revealed all of these three solvents to have less than ideal solubility (activities −0.91, 0.43, 0.02, respectively), consistent with an enhanced degree of solute/solute interactions and solute clustering within the solution structure. However, desolvation in water was found to be more energetically favorable than EtOH due to the hydrophobic nature of the PABA molecule, consistent with its low aqueous solubility and also its higher solubility in protic solvents such as EtOH compared to aprotic solvents such as AcN.

**Figure 14:**
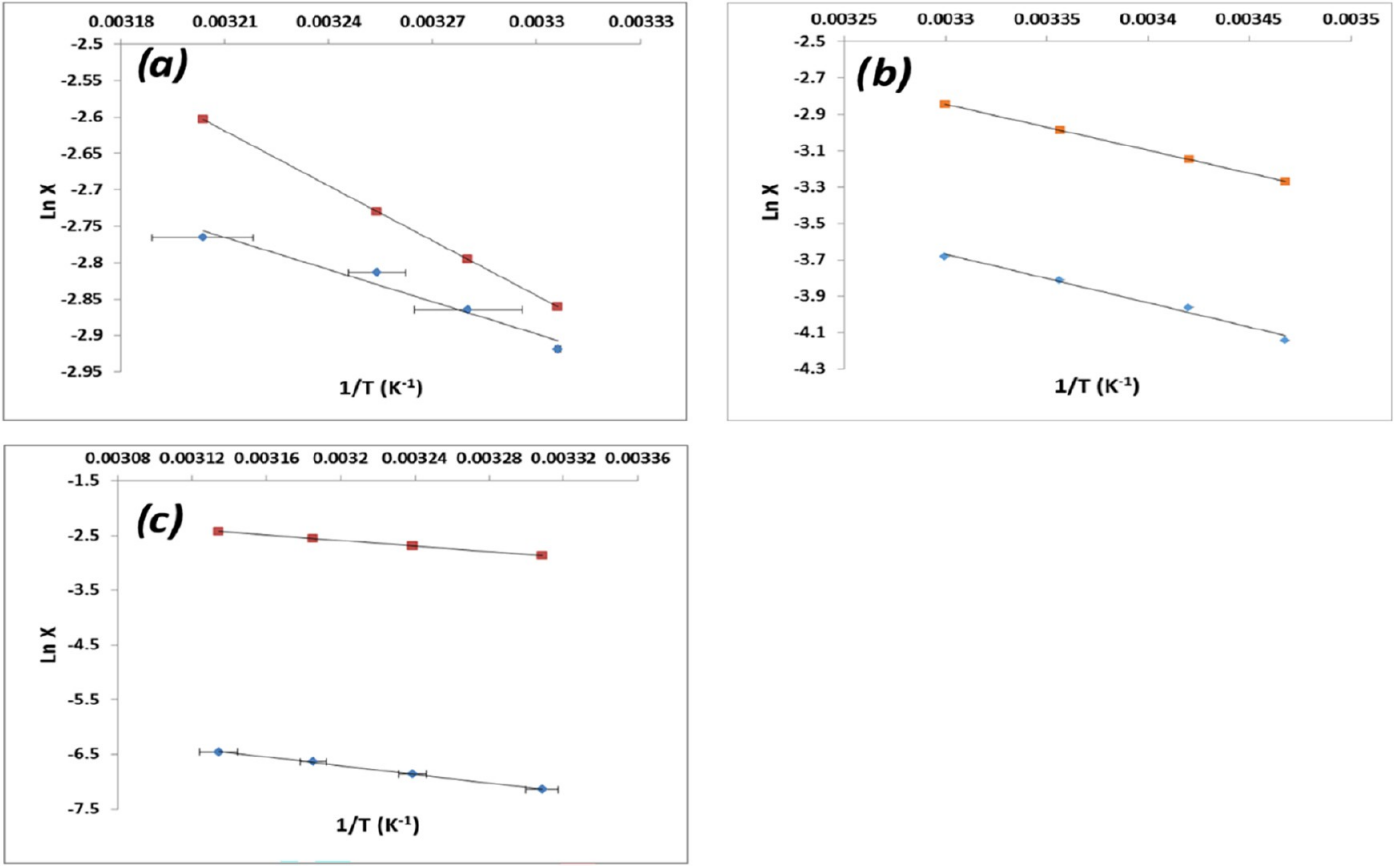
van’t Hoff analysis of PABA solubility (blue) from polythermal analysis vs the ideal solubility (red) in three solvents: (a) ethanol, (b) acetonitrile, and (c) water (data derived from^7^).

### 4.2 Solute Solvation

Intermolecular grid search methods^5,66^ have been used to examine the intermolecular chemistry associated with the solvation of PABA molecules, revealing the predicted solvation shell structures and solvation energies (−29.86, −26.3, and −19.63 kcal mol^–1^, respectively) for 3 solvent systems, namely, EtOH, AcN, and water, as presented in Figure 15, to be consistent with their measured solubilities.^5^ Close examination of the intermolecular interactions reveals that the protic solvent EtOH solvates the solute molecule through strong interactions with both the aromatic ring through vdW interactions with the alkyl group and with the carboxylate group via H-bonding. In contrast, aprotic AcN was found to solvate to a much lesser degree, forming quite limited interactions with the COOH group compared to EtOH, probably reflecting the formation within the solution phase of α-PABA’s OH···O H-bonding dimers (synthon Aα), providing restricted opportunities for hydrogen bonding and hence being consistent with its easier crystallizability in this solvent. As might be expected, the hydrophilic water molecules were found to provide very limited solvation of the overall surface area of PABA, with most interactions being focused on the COOH group with limited interactions with both the amino group and the hydrophobic phenyl ring, which overall reflect its much lower aqueous solubility.

**Figure 15:**
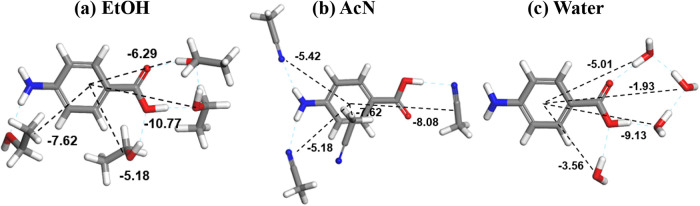
Solvation shell structures of the PABA molecule with three solvents: (a) EtOH, (b) AcN, and (c) water, calculated using molecule–molecule grid searching (data derived from^5^). Note that the solvation shell structures were obtained from up to 10 solvent molecules within the first shell sphere^8^ as beyond this, solvent/solvent interactions can become predominant.

### 4.3 Solute Clustering

A more extensive and detailed analysis of the likely intermolecular interactions associated with solute solvation and prenucleation solute clustering has been examined using the COSMO-RS^67^ approach^29,35^ in order to better understand the polymorph direction process. In this, the expected relative populations for the energetically top-ranked synthons (Aα, Bα, and Aβ, Bβ) for the α- and β-forms in solution over a wider range of solvent systems (Figure 16) have been compared. These studies revealed that synthon Aα, associated with OH···O H-bonding interactions associated with carboxylic acid dimer formation, would be expected to be dominant for all solvents examined with the exception of aqueous solutions where π–π interactions between the aromatic rings were expected to be much more prevalent, notably revealing that the H2H (synthon Bα) and H2T (synthon Aβ) interactions were more stable. Examination of the calculated surface charge distributions for these synthons also revealed the π–π interaction synthons (Bα, Aβ) to have a greater polar surface area compared to the OH···O H-bonding synthons (Aα, Bβ). Overall, these studies indicated that the β-form would be expected to be the more likely polymorph to crystallize from aqueous solutions, while the α-form would be expected to be the preferentially crystallized from most of the other solvents studied.

**Figure 16:**
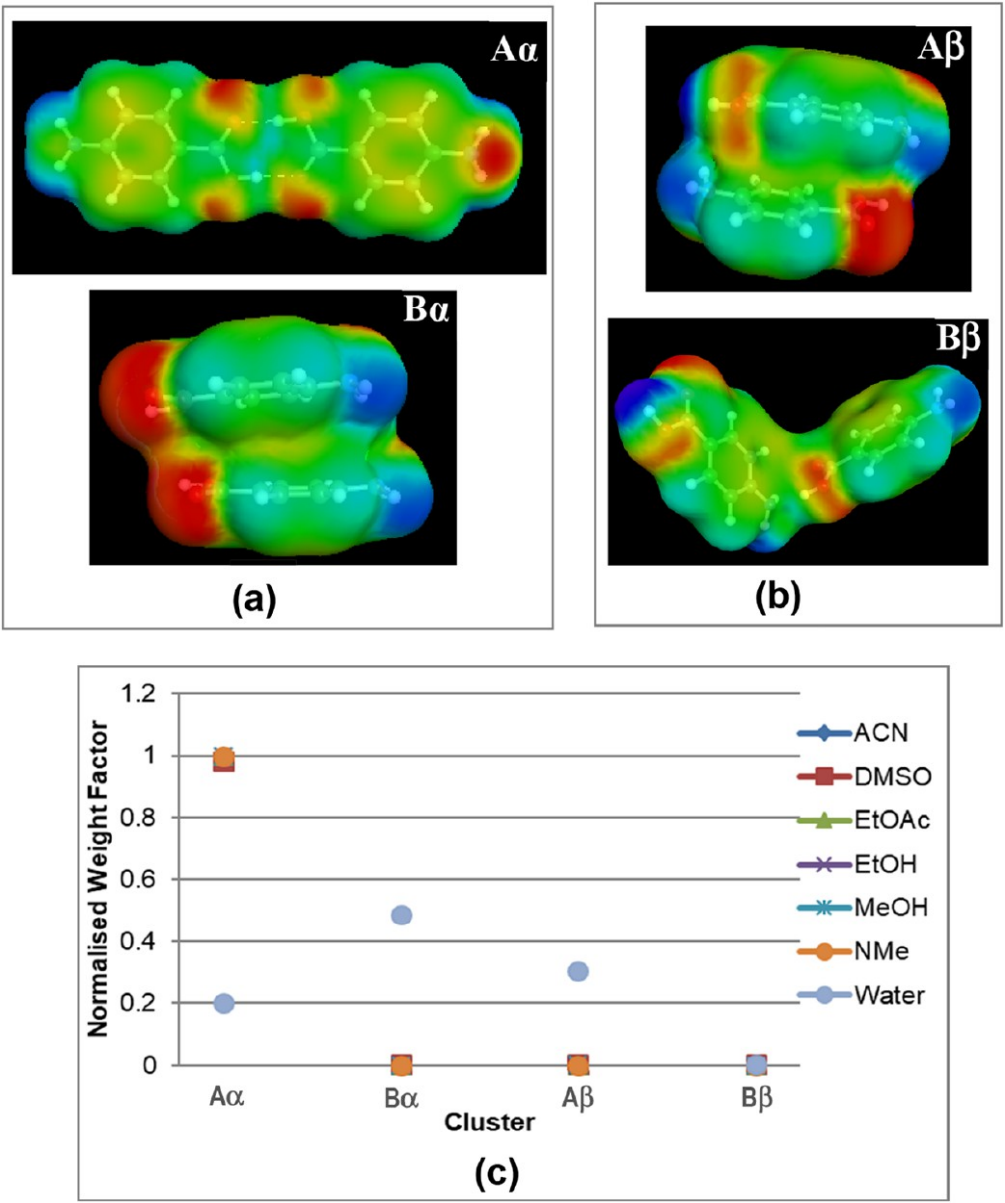
Surface charge density distributions of top two dimer building blocks derived from the (a) α-form and (b) β-form PABA crystal structures, calculated using COSMO-RS based on DFT modeling within a solvent continuum environment and (c) calculated populations of dimers (Aα, Bα, Aβ, Bβ) in seven solvents (AcN, DMSO (dimethyl sulfoxide), EtOAc (ethyl acetate), EtOH, MeOH (methanol), NMe (nitromethane), and water) (data derived from^29,35^ ).

The pathway for PABA transformation from its solvated structure in the solution phase through to its 3D crystalline solid form has been examined through an integrated study encompassing a combination of MD simulations, intermolecular synthon analysis, and Fourier transform infrared spectroscopy (FTIR) in order to characterize the propensity of the incipient bulk synthons that would be important in the crystallization of the two polymorphic forms within the solution state. The MD results,^2^ as shown in Figures 17–19, revealed the presence of intermolecular interactions between the carboxylic acid (synthon Aα) in solution as well as NH···O H-bonding (synthons Cα, Dβ), π–π (synthons Bα, Aβ), and the aromatic displaced ring van der Waals interactions (synthons Dα, Cβ). The singular OH···O and carboxylic acid H-bonding dimer (double OH···O) interactions were more easily found in the AcN solutions than in the EtOH and then H_2_O solutions (Figure 17a,b). As shown in Figure 18a,b, the NH···O (in both α- and β-forms) H-bonding interactions were found to be stronger than the OH···N (only in β-form) H-bonding interactions between amino and carboxylic groups for all three solvents, with the former being quite similar between AcN and EtOH but significantly lower in the aqueous solutions. The latter was also found to be similar in AcN and EtOH solutions, but only fractionally lower in the aqueous solutions. Interestingly, while both the α- and β-forms were found to present strong π–π stacking interactions between the phenyl rings (Aα and Bβ synthons), fewer such interactions were found with the MD studies in AcN and EtOH solutions compared to those in aqueous solutions (Figure 19a,b).

**Figure 17:**
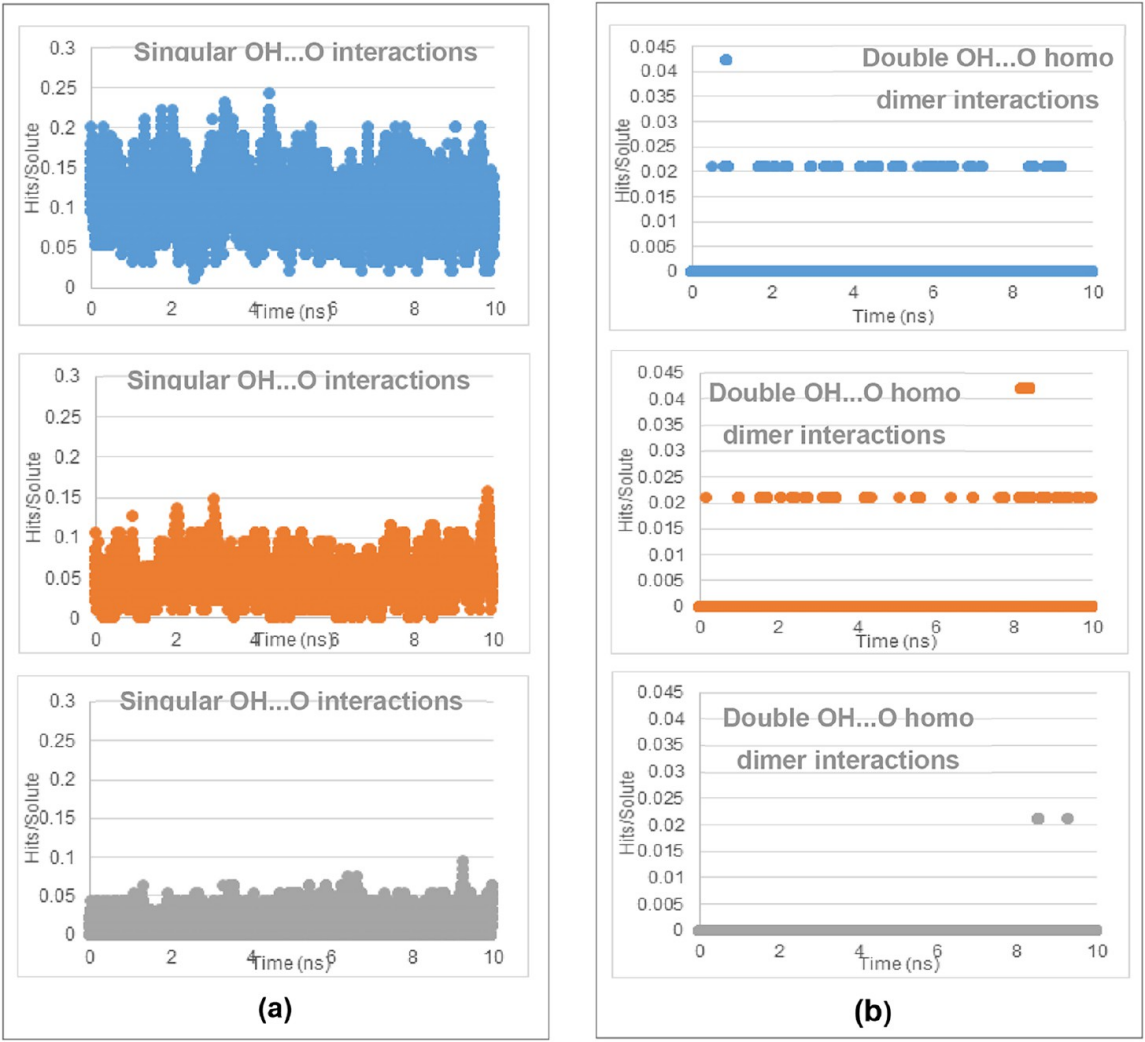
Number of hits found from searching within the MD trajectory files of (a) the singular OH···O interactions and (b) the double OH···O classic homo dimer interactions of PABA in 0.1 g mL^–1^ solutions: AcN (blue), EtOH (orange), and H_2_O (gray) over a 10 ns simulation. Hits are normalized relative to the amount of solute molecules per simulation (data derived from^2^).

**Figure 18:**
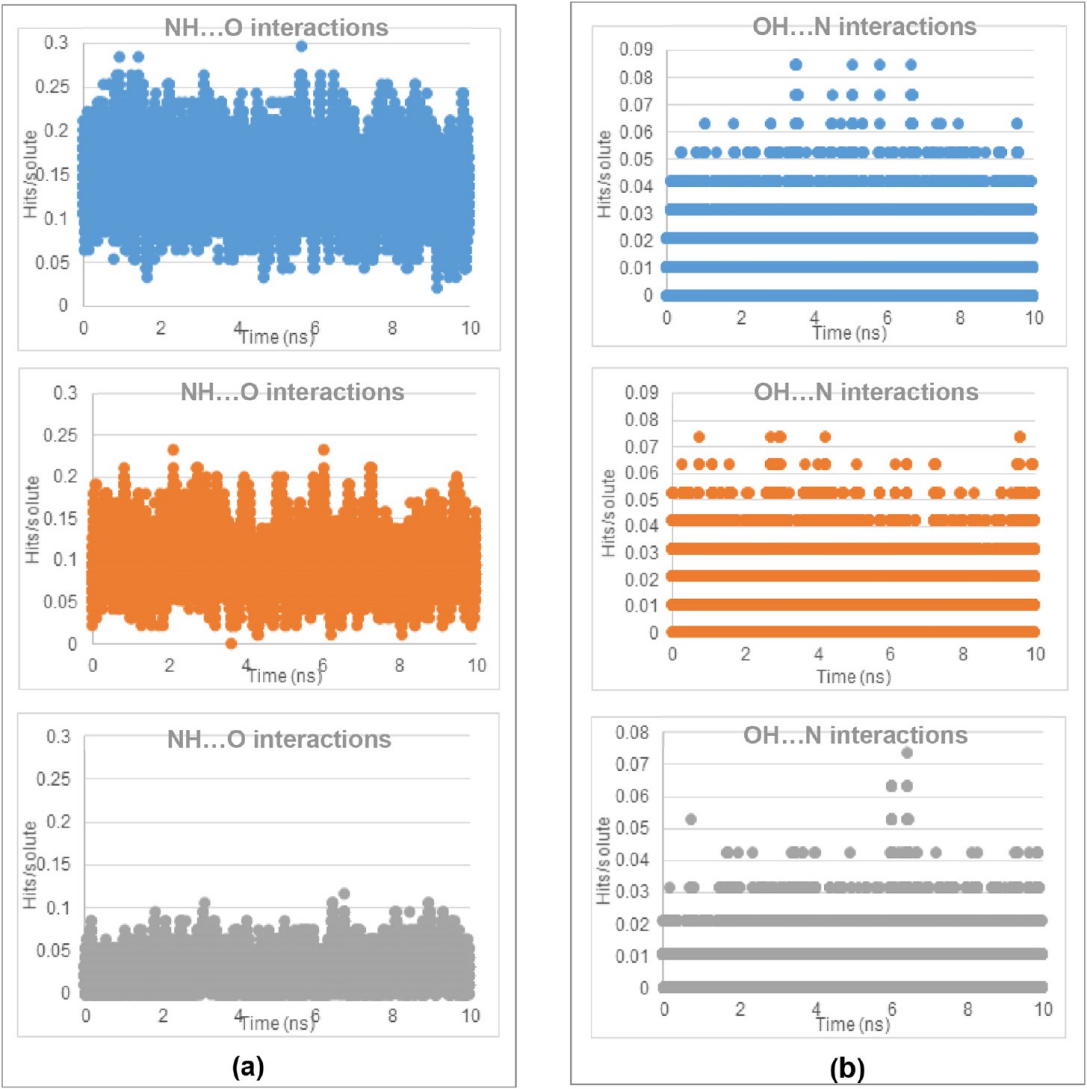
Number of hits found from searching within the MD trajectory files of (a) the singular NH···O interactions and (b) the singular OH···N classic homo dimer interactions of PABA in 0.1 g mL^–1^ solutions: AcN (blue), EtOH (orange), and H_2_O (gray) over a 10 ns simulation. Hits are normalized relative to the amount of solute molecules per simulation (data derived from^2^).

**Figure 19:**
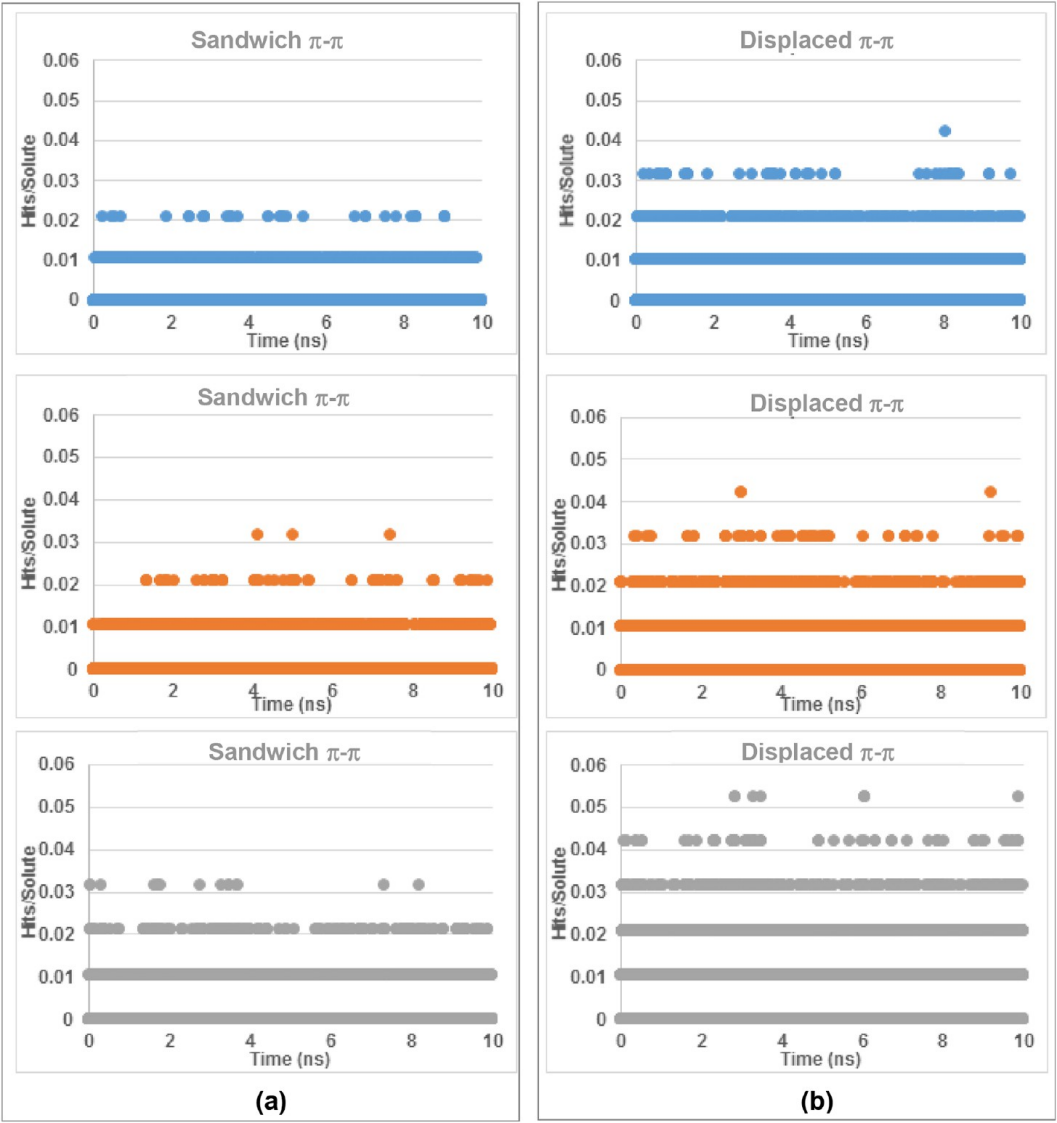
Number of hits found from searching within the MD trajectory files of (a) the sandwich π–π interactions and (b) the displaced π–π interactions of PABA in 0.1 g mL^–1^ solutions: AcN (blue), EtOH (orange), and H_2_O (gray) over a 10 ns simulation. Hits are normalized relative to the amount of solute molecules per simulation (data derived from^2^).

The MD analysis was supported by solution and solid-state FTIR studies, which revealed the spectra in the EtOH and AcN solutions to have a closer resemblance to that found in the analysis of the α-form when compared to the β-form.^2^ The solution FTIR data were also consistent with the presence of both solvated monomers and carboxylic acid dimers (synthon Aα) within the solution phase. Unfortunately, in this study strong solvent absorption precluded FTIR characterization within aqueous solutions.

Overall, the grid search, COSMO-RS and MD simulations, together with the FTIR analysis, have been found to be consistent with a model in which the hydrophobic aromatic rings would tend to close-pack by π–π interactions (synthon Bβ) within a hydrophilic protic solvent with low dispersive interactions such as water (Figure 19a,b). Additionally, the fact that protic EtOH, in comparison with aprotic AcN, tends to solvate PABA solute molecules more equantly and strongly in 3D,^2^ supports the experimental observation of PABA's greater crystallizability in AcN compared to EtOH, while the FTIR analysis highlights the high similarity between the solute-phase data and the α-form structure (synthon Aα) in both EtOH and AcN solutions when compared to the β-form.

### 4.4 Solution Metastability and Cooling Rate–Nucleation Onset Dependence

Polythermal analysis^2,7^ of the cooling rate (*q*) dependence of the crystallization onset points (*T*_c_) as a function of the solvent has been used to characterize the metastable zone width (MSZW) and, through this, to probe the balance between the relative rates of supersaturation generation and the nucleation onset. The relative crystallizabilities for the three solvents (Figure 20a–c, lower plots) highlight their distinctly different behavior with water having a much less dependence on the solution cooling rate compared to EtOH and AcN, respectively, and overall with the crystallizability rate-limiting parameters being in the order of (kinetics-driven) EtOH > AcN > water (thermodynamics-driven). As an example, the polythermal data for the protic solvent EtOH as a function of the cooling rate present a marked difference in the slope with respect to the ideal solubility line, as shown in Figure 20d. The slightly less than ideal behavior at equilibrium solubility can be seen to become greater than ideal with the increasing cooling rate, solution undercooling, and hence supersaturation. Qualitatively, by taking the slope as an indicator of the enthalpy of dissolution (Δ*H*_diss_), Δ*H*_diss_ was found to decrease from ideal solubility (2500 J mol^–1^) to 488 J mol^–1^ (1.0 °C min^–1^) as the EtOH solution undercooled. This decrease in crystallizability with the increased cooling rate can be related to the high solubilization of the solute in this solvent with the higher cooling rates (Figure 20e) of 1.2–1.6, facilitating access to a much wider range of supersaturations and hence nucleation cluster sizes across the solute concentration range when compared to the lowest cooling rates (1.05–1.10).

**Figure 20:**
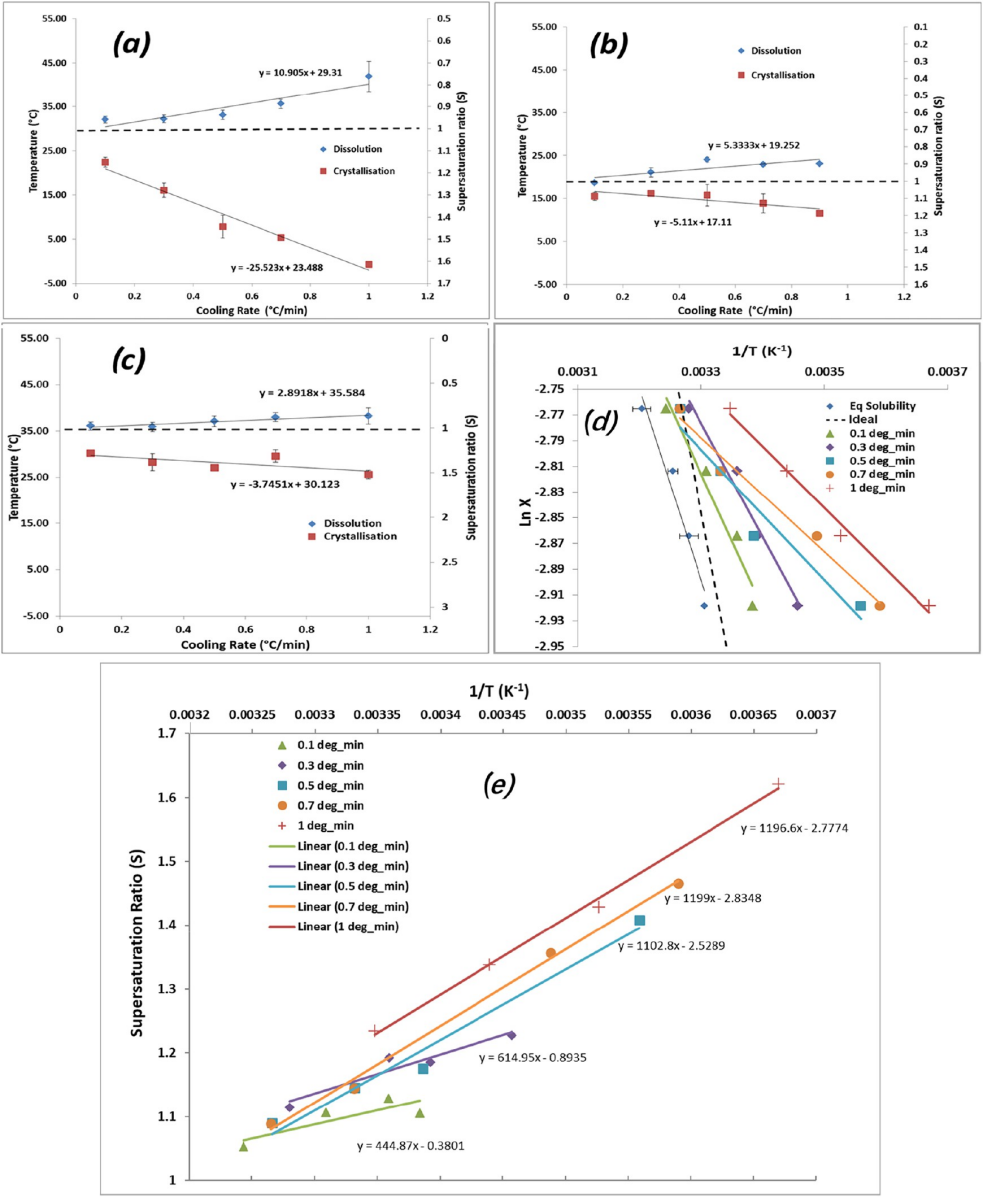
Dissolution and crystallization temperatures of PABA in (a) EtOH, (b) AcN, and (c) water under various cooling rates for the study of its crystallizability and (d) crystallization temperatures of PABA in EtOH with van’t Hoff coordinates, showing a marked difference in the slope with respect to the ideal solubility line (---) and (e) supersaturation ratios in van’t Hoff plotting (data derived from^2,7^ ). Note that panels (d) and (e) are not equilibrium van’t Hoff plots but as such simply kinetic plots of the data using van’t Hoff coordinates for gaining better process understanding, e.g., the impact of undercooling on the range of supersaturation available for crystallization, for which this approach is somewhat analogous to the use of reaction quotient nomenclature in comparison to equilibrium constants in reaction chemistry.

These experimental crystallizability observations also support the solvation energy calculations (Figure 15) and the MD results (Figures 17–19); in particular, OH···O (synthon Aα) interactions are stronger in AcN than in EtOH (Figure 17a,b) due to the latter’s much stronger solvation. In contrast, in aqueous solutions, the solvation was found to be much less, reflecting the fact that the hydrogen-bonded interactions were found to be much lower than those for AcN and EtOH.

### 4.5 Nucleation Kinetics and Mechanisms

The polythermal analysis using the ln *u*_c_ (relative critical undercooling) vs ln *q* plot for EtOH, AcN, and aqueous solutions as a function of solute concentrations is shown in Figure 21, with the kinetic analysis based on the KBHR approach^68,69^ summarized in Table 9. Examination of the data from the “rule of three” analysis^69^ reveals that the nucleation mechanisms in AcN, EtOH, and aqueous solutions were mostly found to be instantaneous (the slope of these plots <3) (Table 9, column 2), and consistent with a two-step nucleation mechanism,^7^ except for the lower solute concentrations for EtOH, AcN, and H_2_O (Table 9), where it was found to be progressive (the slope of these plots >3). Among the instantaneous cases, EtOH was found to be “more instantaneous” than AcN and water, reflecting their respective solubilities and crystallizabilities.^7^ However, the slopes of these regressions in water were found to be 2.13–2.41 (much closer to 3), indicating that PABA should nucleate much more readily in aqueous solutions. Conversely in EtOH and AcN, putative nuclei seem to be stable for longer time periods, consistent with the larger solution undercoolings observed for these solvents.^7^ Isothermal nucleation kinetic analysis as a function of the solvent type and solution concentration reveals the interfacial tension (γ_eff_) and cluster size (r*) in ethanol to be lower than those in water (Table 9, columns 4 and 5).

**Figure 21:**
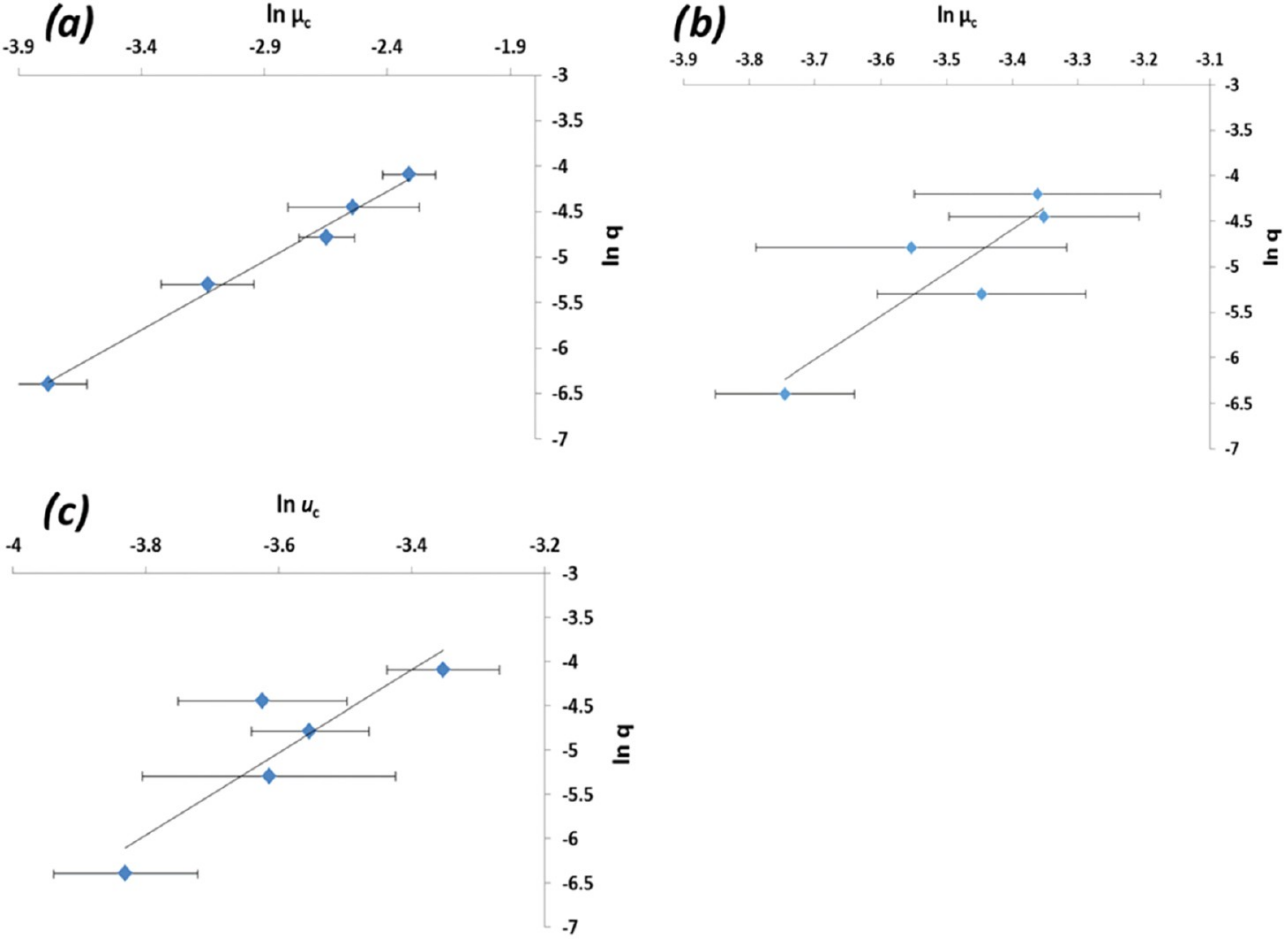
(a) Plots of *q* vs *u*_c_ in ln–ln coordinates for PABA in ethanol at a concentration of 170 g kg^–1^; (b) in acetonitrile at a concentration of 54 g kg^–1^; and (c) in water at a concentration of 6 g kg^–1^ (data derived from^7^).

**Table 9:** Nucleation Kinetics Analysis as a Function of Concentration for α-PABA in Ethanol, Acetonitrile, and Water from the Slope and Intercept of the Linear Fit to *q* vs *u*_c_ in ln–ln Coordinates^a^

concentration (g kg^–1^)	slope of ln *q* vs ln *u*_c_	nucleation mechanism	γ_eff_ (mJ m^–2^)	r* (nm)	Δ*T*_c_ range (°C)
**Ethanol**					
150	3.69	progressive	2.71^b^	0.3–0.5	16.1–25.8
160	3.25	progressive	1.79^b^	0.2–0.4	14.2–29.8
170	1.68	instantaneous	N/A	N/A	8.3–31.4
180	1.76	instantaneous	0.85^c^	0.7–2.0	7.3–21.5
190	1.56	instantaneous	N/A	N/A	5.6–17.1
200	1.62	instantaneous	1.31^c^	0.5–0.9	4.2–13.9
**Acetonitrile**					
54.0	4.77	progressive	1.13	0.6–0.8	6.8–10.0
64.8	1.56	instantaneous	N/A	N/A	3.8–7.8
75.6	1.61	instantaneous	N/A	N/A	3.0–4.9
86.4	1.99	instantaneous	N/A	N/A	2.2–5.5
**Aqueous Solutions**					
6	4.53	progressive	1.95	0.5–0.8	6.6–10.6
8	2.41	instantaneous	N/A	N/A	5.4–10.0
10	2.39	instantaneous	N/A	N/A	3.5–7.2
12	2.13	instantaneous	N/A	N/A	2.6–6.2

a The data are derived from ref 7.

b Calculated from polythermal KBHR analysis.^7^

c Calculated from isothermal analysis.^27^

### 4.6 Additive Control of the Nucleation

The control of the nucleation process from ethanolic solutions in the presence of tailor-made additives (TMAs) including 4-amino-3-methoxybenzoic acid (AMBA) and 4-amino-3-nitrobenzoic acid (ANBA) has been examined^9^ (Figure 22a), all TMAs containing a carboxylic acid structural group in order to investigate the effect of these additives on the nucleation process. Calculation of the dimer interaction energies found that the three TMAs selected (ANBA3, AMBA2, and AMBA3) interacted more strongly with PABA than PABA did with itself, suggesting that these TMAs would be likely to compete with PABA within the solution phase to form H-bonding dimers (PABA:ANBA3, PABA:AMBA2, and PABA:AMBA3) through interactions with the important synthon Aα (Figure 11) instead of that with the host PABA:PABA dimer. The solvation calculations of these four dimers (Figure 22a) confirmed that the three PABA:TMA dimers are more strongly solvated than the PABA:PABA dimer, with the strongest one being PABA:ANBA3 (Table 10), suggesting that this dimer might be more stable within the solution phase and hence harder to desolvate for subsequently forming the full range of PABA intermolecular interactions in the solid-state and hence, through this, making PABA nucleation more difficult.^9^ Examination of the interaction geometries of the four dimers (Figure 22a) found that for PABA molecules interacting with PABA:PABA, PABA:AMBA2, and PABA:AMBA3 dimers, synthon C was found to be the strongest intermolecular interaction (Figure 22b1, b3, b4), which involves the carboxylic acid groups of the dimer interacting with the amino group of the PABA molecule, directly linking with the preassembly mechanism of PABA. In contrast, synthon C was not found to relate to the strongest intermolecular interaction with the PABA:ANBA3 dimer and a PABA molecule (Figure 22b2). Instead, the amine group of the interacting PABA molecule was found to interact with the nitro group of ANBA3,^9^ directing the formation of strong non-crystallographic synthons (Figure 22b2).

**Figure 22:**
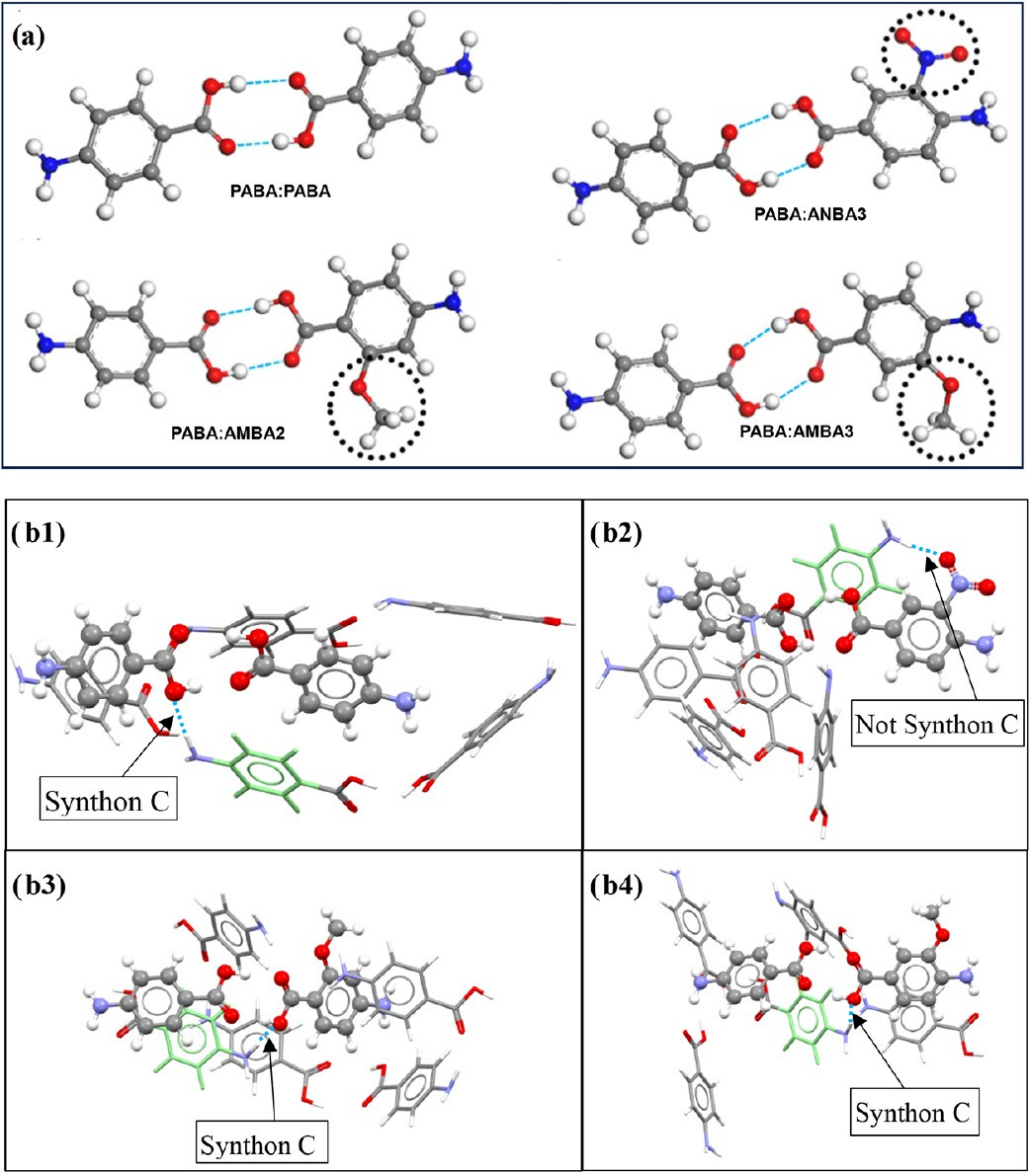
(a) Most favorable dimer interactions of PABA:PABA, PABA:ANBA3, PABA:AMBA2, and PABA:AMBA3, calculated from the intermolecular grid search (H-bonding is shown by the blue dashed lines and additional functional groups of TMAs are highlighted in the dotted circles). (b) Interaction clusters of 4 PABA molecules around the calculated most energetically favorable (b1) PABA:PABA, (b2) PABA:ANBA3, (b3) PABA:AMBA2, and (b4) PABA:AMBA3 dimers. Dimers are shown with the ball and stick style. PABA probe molecules are shown with the capped stick style. The strongest PABA intermolecular interactions with each dimer are shown with PABA molecules with light green cyclic rings. Blue dashed lines represent intermolecular hydrogen bonding for the strongest PABA interaction with the dimer (data derived from^9^).

**Table 10:** Solvation Energies of 20 EtOH Molecules Interacting with PABA:PABA and PABA:TMAs and MZWs for PABA in EtOH Solutions at a Cooling Rate of 0.5 °C min^–1^ without and with the Presence of TMAs

		PABA + TMA		
compound	pure PABA	ANBA3	AMBA2	AMBA3
solvation energy (kcal mol^–1^)	–58.7	–76.0	–60.4	–73.5
MSZW (°C)	18.2	41.5	28.8	23.7

Experimental screening using polythermal analysis confirmed the ability of these TMAs to lower the crystallization temperature of PABA in ethanol solution (Figure 23), consistent with the modeling findings. ANBA3 was found to have the strongest PABA nucleation inhibition capabilities due to its meta-position nitro group having a large effect on the ability of PABA molecules when bound to it to form the key synthonic interactions, notably synthon C, needed to assemble the extended intermolecular cluster needed to nucleate the crystalline phase.^9^ Examination of the crystallizability, solution thermodynamics, and nucleation kinetics of PABA:ANBA3 revealed large increases in the steady-state MSZW and solvation energies (Table 10) in the presence of TMAs, albeit with little changes in the solute solubility (Figure 23).^9^ Significantly, the nucleation pathway in the presence of the additives was found to change from instantaneous in pure PABA to progressive nucleation in the presence of TMA,^9^ consistent with the predictive modeling screening outcomes. Overall, the study revealed that the TMAs can widen the MSZW via depressing the crystallization onset point, leading to an increase of solution supersaturation for nucleation and a concomitant decrease in the cluster size and, through this, switching the nucleation pathway from an instantaneous to a progressive mechanism.^9^

**Figure 23:**
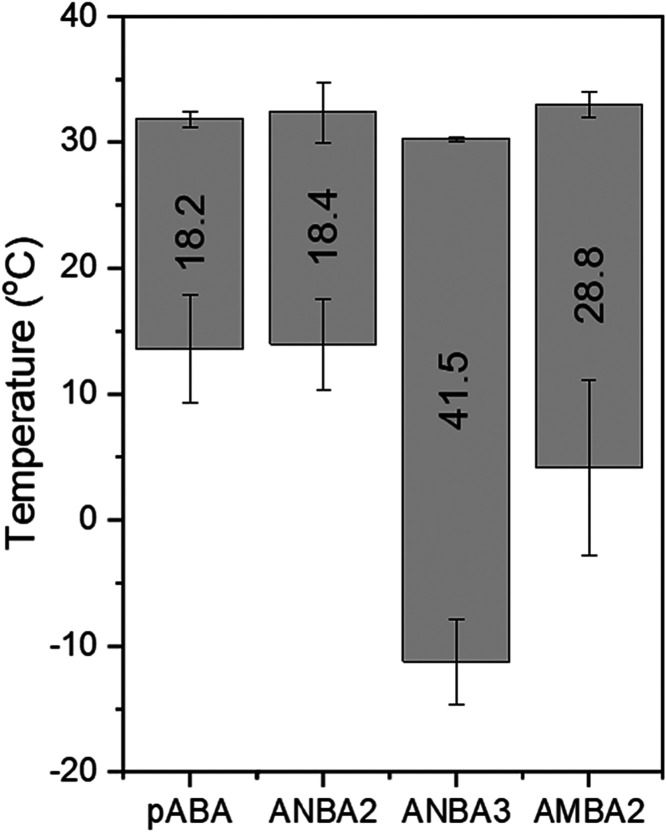
Floating column chart displaying *T*_c_ (bottom of columns) and *T*_diss_ (top of columns) values determined for PABA in an ethanol solution at a cooling rate of 0.5 °C min^–1^ with and without the presence of TMAs. Error bars show standard deviations of results. Numeric values within columns represent the dynamic MSZW in °C (data derived from^9^).

This study is consistent with the studies by Black et al.^70^ who also examined the effects of these additives on the nucleation and growth kinetics of PABA crystals for both α- and β-forms, revealing a much larger impact on the α-PABA growth kinetics but with a similar scale of slowing down the nucleation of both forms, hence leading to the nucleation and growth of the β-form under the conditions normally suitable for the α-form. This possibly reflects the selected additives attaching strongly to aromatic rings, hence disrupting the formation of π–π stacking in α-PABA and, through this, promoting the growth of the β-form whose development is largely controlled by the tetrameric assembly of a ring of alternating NH···O and OH···N hydrogen-bonding interactions rather than π–π stacking.^9^

### 4.7 Nucleation Pathway

Characterization of the solute clustering in PABA solutions in EtOH has been examined using small-angle X-ray scattering (SAXS)^27,37^ with the results of scattered intensity (*I*_(q)_) as a function of *q* summarized in Figure 24, highlighting the presence of solute clusters within the solutions even when undersaturated.

**Figure 24:**
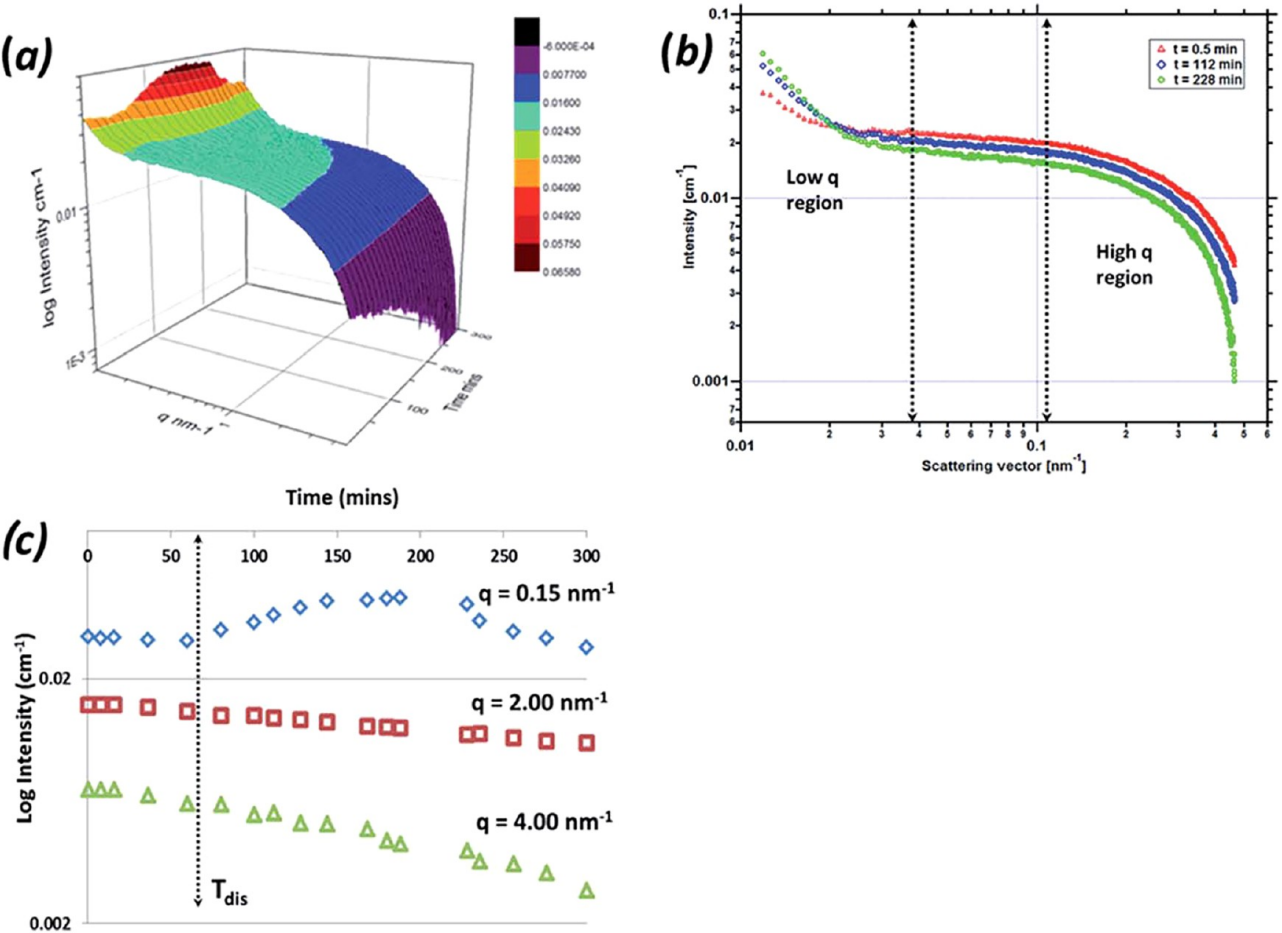
(a) 3D log–log plot of absolute *I*_(q)_ vs *q* as a function of time during a polythermal cooling crystallization at 0.1 °C min^–1^; (b) log–log *I*_(q)_ vs *q* at the start of the cooling profile (0.5 min), the middle of the cooling profile (112 min), and at *I*_max_ for the low *q* region (228 min); and (c) *q* progression in log coordinates throughout the cooling process for three regions of the *q* space; 0.15, 2, and 4 nm^–1^ (data derived from^27^).

A more detailed examination of the temporal evolution of the SAXS data at a low scattering vector (*Q*) revealed an increase in both the numbers of solute clusters and their sizes (>40 nm) during the solution cooling process, hence confirming the formation of solvent clusters within the solution phase. At the low *Q* region, SAXS data was not found to be very well differentiated due to the intensity being cut off by the beam-stop, limiting the low *Q* region beyond 0.1 nm^–1^, hence restricting the scattering in this region from larger aggregates in the region of 10–40 nm. The behavior of such clustering effects is consistent with previous studies on, for e.g., glycine,^71^ citric acid,^72^ and l-isoleucine.^73^

The complementary analysis of the SAXS data at high *Q* reveals the effect of solution desupersaturation during the cooling process, providing some evidence for the formation of small intermolecular cluster structures (ca. 1–2 nm) within the size range that would be well matched to the overall dimensions of the carboxylic acid dimer (synthon Aα).

Figure 25 schematically shows a tentative nucleation pathway for PABA crystallization from EtOH solutions drawing together the structural information obtained from the analysis of both the modeling and experimental data. The overall nucleation pathway indicates the development of nanoscale assemblies from large disordered liquid-like nanoclusters (dimers and monomers) in undersaturated conditions. These clusters then grow in size and structural ordering under supersaturated conditions. Their mass fractal dimension increases as does the interfacial smoothness and faceting at the crystal/solution interface and, through this, leads to the formation of the crystalline phase (nuclei) by further growth.

**Figure 25:**
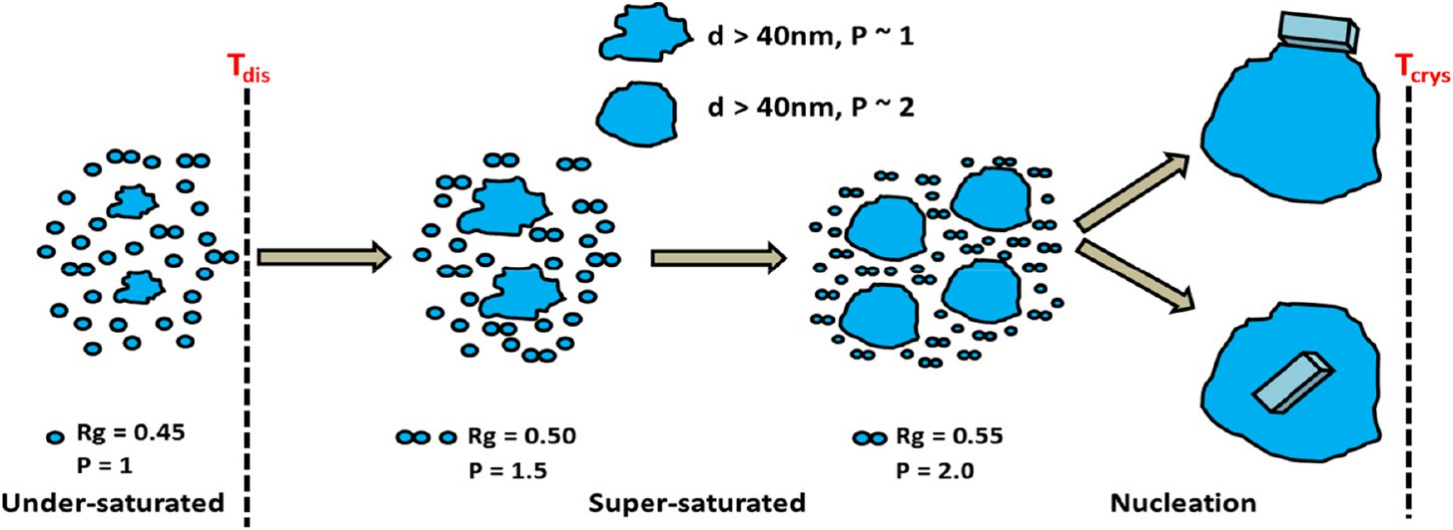
Tentative structural schematic of the α-PABA nucleation pathway from EtOH solutions, based on the modeling and experimental evidence obtained so far, indicating large liquid-like clusters surrounded by a population of monomers and dimerized PABA molecules. Note that the pathway for β-PABA nucleated in water can have different parameters. *Rg* is the radius of gyration which is related to the cluster size (nm) and *P* is the fractal dimensionality which is related to the interface structure where a higher value infers a more ordered interface (data derived from^2,37^ ).

However, the SAXS studies^27^ were of necessity restricted to high-solubility solvent systems, and at the higher solute concentrations, mindful SAXS characterization of solute clusters can be problematic contrast-wise for lower solubility systems, as the SAXS measurements require a good contrast between the aggregated and continuum phases. This restriction hence precluded SAXS studies over a range of solute concentrations in EtOH solutions as well as for studies of the same in AcN and aqueous solutions. Thus, the data, to date, have been limited to a single solvent system at solute concentrations where instantaneous nucleation and solute clustering might be expected. It is obviously an open question as to whether such solute clusters are also formed at lower solute concentrations where progressive nucleation might be more likely, and hence, further work is needed in the future to examine this aspect.

## 5 Crystal Growth

### 5.1 Observed Crystal Morphologies

Analysis of experimental crystallization data reveals that the observed morphology^27,36^ of α-PABA often demonstrates a much more needle- or lath-like external morphological form than that predicted (Figure 13). This is not unexpected due to the different growth conditions for the morphological predictions (an equilibrium situation, i.e., zero supersaturation) and the experimentally observed morphologies (supersaturated conditions). In contrast, the β-form has been found to crystallize with a prismatic equant morphology in good agreement with predictions.^43^

In terms of the α-form morphology, the bonding propensity of the crystallization solvents has been found to significantly change its external morphology following crystal growth. In particular, hydrophilic solvents such as EtOH, AcN, and water tend to have strong interactions with the crystal habit faces whose growth processes encompass dominant H-bonding interactions such as the {10–1} prismatic faces. In contrast, the more hydrophobic solvents tend to bind to the {01-1} capping faces, where aromatic interactions play a much more important role in their growth. This repulsion of water from PABA’s aromatic ring together with their high affinity for the polar carboxylic acid groups as identified from the grid-based search molecular modeling^5^ can result in very thin α-PABA crystals and also the appearance of the β-form under certain conditions from this solvent only. The experimental studies of the crystal morphology of α-PABA crystallized from EtOH, AcN, and water solutions revealed that EtOH generally produces α-form crystals (Figure 26a,d) elongated along the needle *b* axis, with their crystallographic habit surfaces being much more clearly defined, when compared to those prepared from aqueous solutions,^7^ while growth from AcN solutions generally produced crystallographically well-defined crystals (Figure 26b), which were also found to be somewhat wider than the crystals prepared from water and usually exhibited shorter needle-like crystals (Figure 26c,d) with much less defined facets.

**Figure 26:**
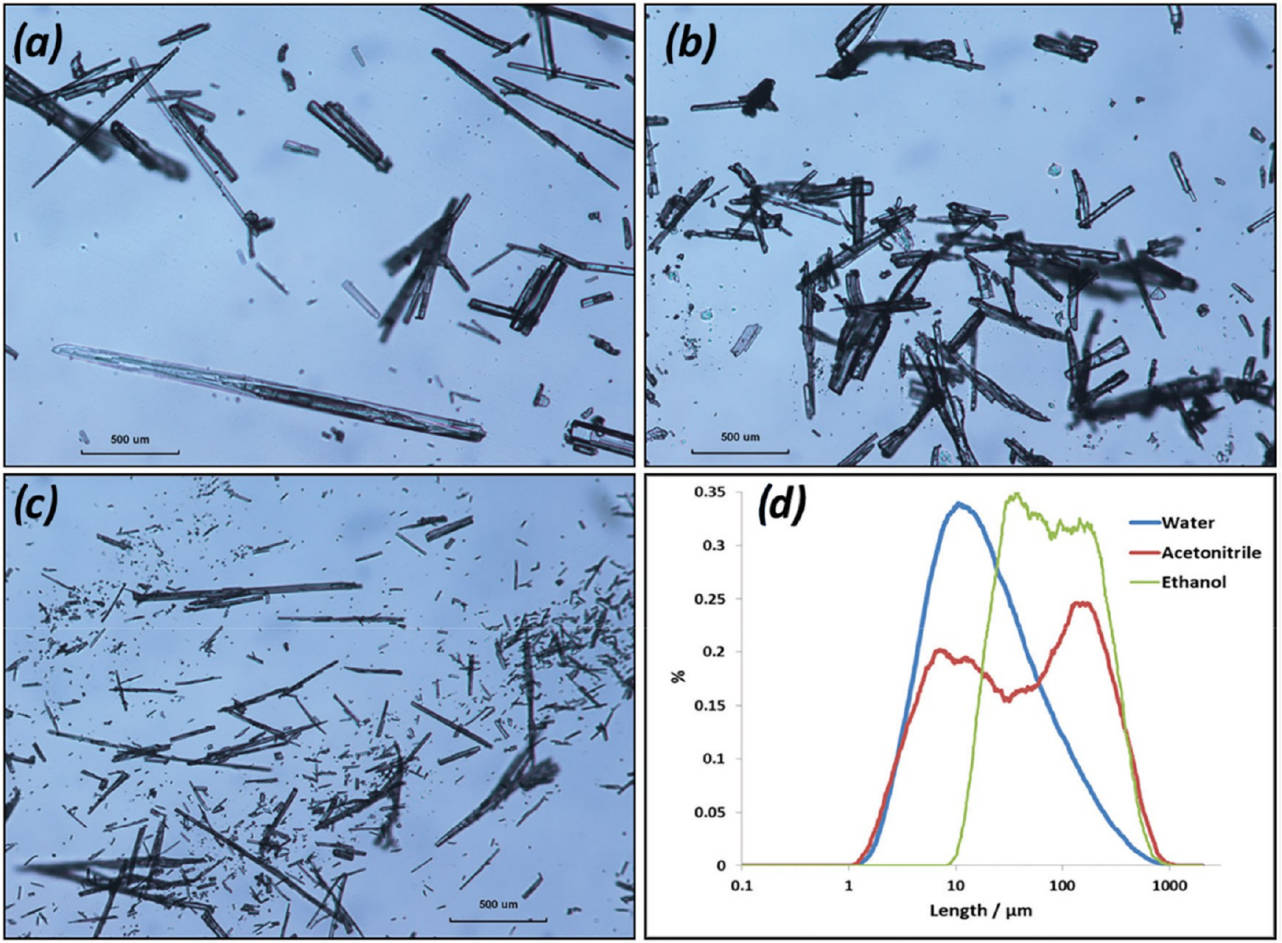
Crystal images crystallized in (a) ethanol, (b) acetonitrile, and (c) water solutions and (d) length distributions of these crystallites from cooling crystallizations at σ = 0.2 using optical analysis (data derived from^7^).

In the similar manner to some selected additives inhibiting the formation of π–π stacking in α-PABA through strong binding to the aromatic rings,^9,70^ the disruptive effect of the more hydrophobic solvent NMe molecules on the growth process has also been observed through crystallization from mixed EtOH/NMe solutions.^34^ In this, grid search modeling (Figure 27) suggests that NMe molecules prefer to interact strongly with the phenyl rings governing the growth of the {110} capping faces (Figure 27a), while EtOH tends to bind most strongly to the COOH group (Figure 27b).

**Figure 27:**
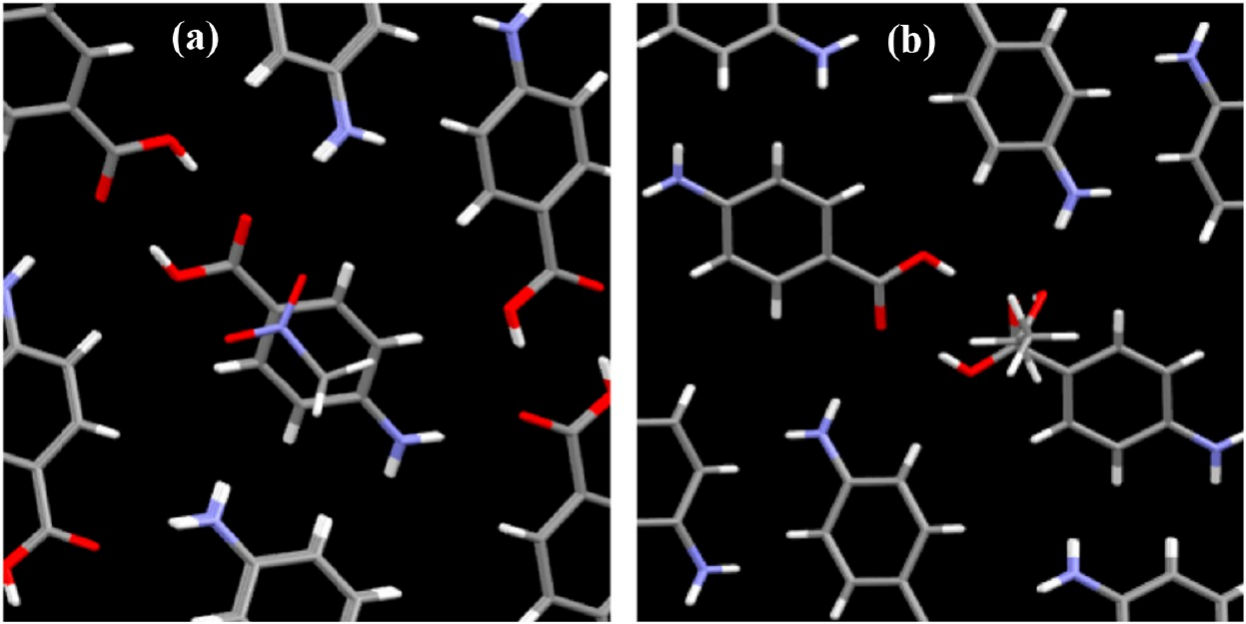
Most favored orientation for a probe molecule of (a) ethanol and (b) nitromethane at the {0 1 −1} surfaces of the α-form of PABA (data derived from^34^).

At high NMe concentrations within the mixed solutions, the α-form crystallization was found to be suppressed, leading to the formation of a new NMe solvate of PABA. The presence of NMe was also observed to lead to significant changes in the crystal habit modification,^34^ as shown in Figure 28. In this, the disruption in the π–π stacking interactions in this solvent system was found to result in a decrease in the aspect ratio of crystals obtained with increasing NMe content within the mixed solution.

**Figure 28:**
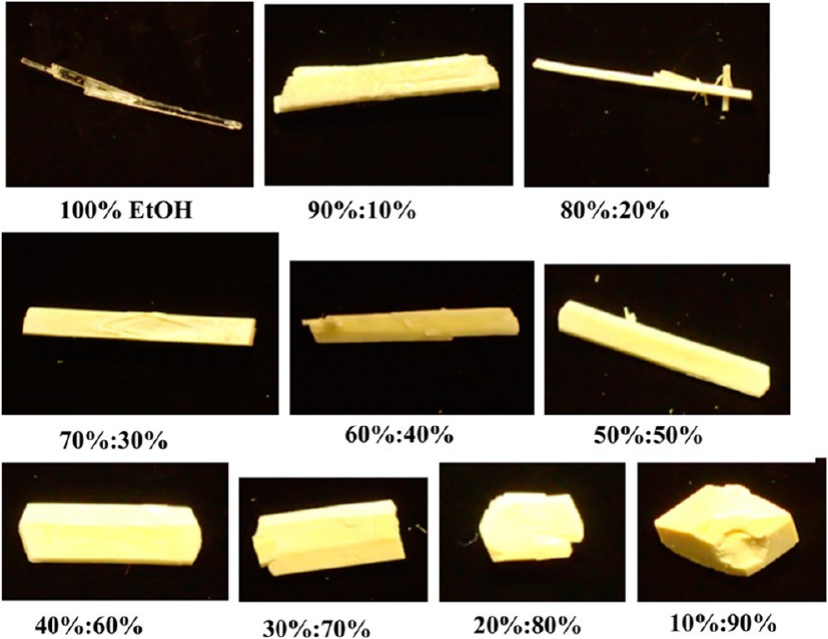
Images of PABA crystallized at σ = 0.1 at 10 °C from pure EtOH up to a 90:10 NMe/EtOH ratio (data derived from^34^).

The habit-modified crystals were also observed to become more faceted with smoother and more defined habit surfaces, effectively seeming to stabilize the normally rougher interfacial growth of the α-form capping face,^27,34,43^ leading to the slower and stable growth of that face.^34^

### 5.2 Crystal Facet Growth Kinetics

Measurement of the growth rates of the α-form normal to the {01–1} and {10–1} faces^27^ as a function of relative supersaturation (*σ*) is shown in Figure 29. The growth rate dependence of the {01–1} capping faces could be fitted with a rough interfacial growth (RIG) mechanism (a coefficient of determination *R* ^2^ = 0.988) across the whole supersaturation range of σ = 0.02–0.20 studied (Figure 29a). In contrast, fitting of the growth kinetics for the lateral prismatic {10–1} face form was found to be consistent with a birth and spread (B&S) growth mechanism (*R* ^2^ = 0.994) (Figure 29b) with a clearly defined dead zone for zero growth for supersaturations less than 0.1. To date, the growth rate for the slowest growing {101} faces has yet to be measured. The different growth behaviors of these two habit faces reflect their distinctly different surface chemistry and intermolecular surface binding, as revealed from surface visualization (Figure 13) and from the analysis of their extrinsic synthonic intermolecular interactions contributing to their attachment energies.^27^ The dominant contributor to the growth of the {10–1} faces was identified as hydrogen-bonding interactions (synthon Aα) along the growth direction involving the COOH and NH_2_ functional groups. However, the potential hydrogen bond between the OH group in protic solvents such as ethanol and the C=O group of PABA exposed on the surface could be expected to lead to quite a strong intermolecular solvent binding with concomitantly slower desolvation at face {10–1}, hence reducing its growth rate (Figure 29). In contrast, the π–π stacking interaction (synthon Bα) was found to be the most important growth-promoting interaction in the growth of the {01–1} faces and to make the main contribution to the growth of these faces, leading to much faster desolvation at the {01–1} face for polar solvents. The reverse trend in more apolar and nonhydrogen bonding solvents could be also expected as seen in the data for the EtOH/NMe system presented earlier, but detailed growth kinetics of this system are yet to be carried out. Overall, the intermolecular interactions with concomitantly higher attachment energies for the {01–1} faces were indicative of the potential for significant growth instability for these faces.^27^

**Figure 29:**
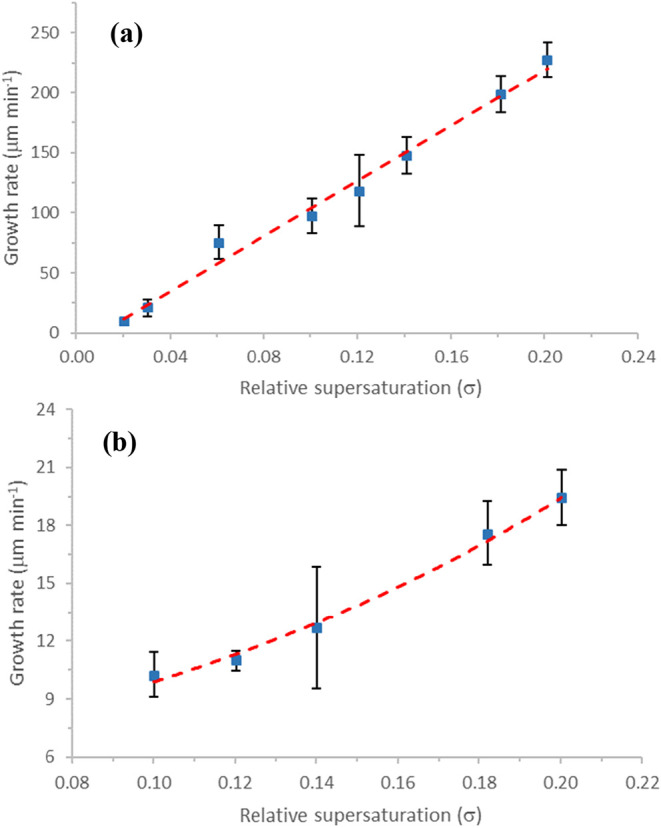
Facet growth rates of PABA in ethanol with error bars and fittings (red dashed lines) of growth kinetics models: (a) face (01–1) fitted (*R* ^2^ = 0.988) with a rough interfacial growth mechanism and (b) face (10–1) fitted (*R* ^2^ = 0.994) with a birth and spread mechanism (data derived from^27^).

Some comparative values of previously published growth rates as a function of solution supersaturation for individual crystal faces for a range of organic compounds in different solvents are shown in Table 11, revealing the growth rates of PABA presented in this work to be within the same order of magnitude of those measured for similar organic systems. As an example, the {01–1} capping and {10–1} prismatic faces of α-PABA were found to have, e.g., the similar growth rate ranges as the {101} capping and {021} prismatic faces of the β-form of l-glutamic acid, albeit with the latter being measured at a higher range of solution supersaturations of 0.28–1.21 compared to 0.1–0.2 for α-PABA.

**Table 11:** Comparative Published Growth Rates for Individual Crystal Habit Faces of Some Organic Crystalline Systems

compounds	σ	range of growth rates (μm s^–1^)	refs
l-glutamic acid (β-form) in water			
{101} faces	0.28–1.21	0.46–4.01	^74^
{101̅} faces	0.28–1.21	0.52–4.13	^74^
{021} faces	0.28–1.21	0.01–0.44	^74^
{010} faces	1.05	0.21	^75^
**paracetamol** in the [110], [010], and [001] direction	0.06–0.26	0.02–0.16	^76^
**ibuprofen** {001} and {011} faces in ethanol/water, ethyl acetate, acetonitrile, and toluene	0.55–1.3	0.04–2.02	^77^
**hexatriacontane** (C_36_H_74_) {110} faces in petroleum ether	0.22–0.70	0.00–2.00	^78^
***n*-octacosane** (C_28_H_58_) {110} faces in petroleum ether	0.01–0.15	0.00–14.00	^79^
**dotriacontane** (C_32_H_66_) {110} faces in m-xylene	1.00–1.50	1.00–3.00	^80^
**stearic acid** (B and C polymorphs) {110} faces in butanone	0.01–0.30	0.00–2.80	^81^
**stearic acid** (B polymorph) {110} faces in decane	0.01–0.40	0.00–0.40	^82^
***n*-docosane** {010}, {112}, {102} and other not indexed faces in *n*-dodecane	0.01–0.05	0.51–9.85	^83^
**methyl stearate** {110} and {11̅0} faces in			^84^
*n*-dodecane	0.30–0.39	0.09–1.13	
kerosene	0.45–0.52	0.01–0.35	
toluene	0.04–0.08	0.02–0.37	
**tolfenamic acid** in EtOH			^85^
form I {100} and {011} faces	0.10–0.70	0.00–0.56	
form II {110} and {020} faces	0.3	0.194 and 0.004	
**PABA** in EtOH			^27^ and this work
{011̅} faces	0.10–0.20	0.16–3.60	
{101̅} faces	0.10–0.20	0.15–0.30	

### 5.3 Direction of the Polymorphic Form

Several studies^2,7,29,36,86^ have been carried out to examine how the crystallization process itself directs the formation of polymorphic forms. NMR spectroscopy and molecular dynamics simulations have been combined to characterize molecular self-association in acetone, ethanol, and aqueous solutions and, through this, understand polymorph-selective crystal nucleation pathways.^86^ The NMR results have revealed that the key intermolecular interactions which stabilize PABA oligomers can be quite solvent-dependent, i.e., the π–π stacking interactions dominate the PABA-water solution, while the hydrogen bonding becomes more dominant in organic solvents such as acetone and ethanol, with both kinds of intermolecular interactions making their contributions to the self-association process for all three solvents. The complementary MD simulations revealed that the oligomers can display a fluxional character with a short life, indicating the growth units most likely to be PABA monomers.^86^ These studies revealed that the presence of symmetric H-bonded carboxylic dimers may be indicative that the simultaneous desolvation of two carboxylic groups would be statistically accessible, hence leading, in turn, to the crystallization of the α-form PABA. In contrast, the β-form would be expected to be formed in solutions where strong solvation of the carboxylic moieties completely suppresses the material’s ability to form the symmetric H-bonded dimers (synthon Aα).^86^ Such findings have also been supported in literature, e.g.,^2,7,29,36^ including the COSMO-RS approach^29^ based on DFT modeling where OH···O H-bonding dimers were found to dominate in all solvents apart from water and where π–π stacking interactions become more favored, consistent with the β-form only crystallizing from this solvent.^29^ MD simulations^7^ reveal a clear evidence for the formation of carboxylic acid dimers in solution but with much more limited evidence for the presence of other key interactions, notably the “finger printing” OH···N interactions (synthon Bβ), which are only present in the β-form. Furthermore, the desolvation of PABA can be expected to be energetically easier in aqueous solutions compared to ethanolic solutions.^7^ The MD studies carried out to date have not been able to discriminate between the H2H (α-form) and H2T (β-form) π–π aromatic ring interactions,^2^ which would perhaps further clarify the nucleation pathway. Grid-based intermolecular solute/solvent search methods^5^ have confirmed that the carboxylic acid dimers (synthon Aα) characteristic of the α-form are very strong interactions when compared to competing solute/solvent interactions and that these lead to dominate the self-assembly process prior to nucleation. The exception to this model is for aqueous solutions where water can disrupt dimer formation, and where at the lower supersaturations, where the nucleation cluster sizes are larger, hence enabling the β-form to more easily crystallize. Additionally, the crystallization of β-PABA has been predicted to require a change in the molecular conformation with a conformational energy penalty of ca. 3 kcal mol^–1^ imposed on its intramolecular packing when compared to the α-form which does not require a significant conformational change, making the latter have higher crystallizability.

## 6 Concluding Remarks

This overview presents some selective research into the crystallization and polymorphic behaviors of *p*-aminobenzoic acid (PABA) from the solution phase, highlighting and interlinking the crystal science underpinning its molecular properties, crystallographic structures, intermolecular crystal chemistry, solution properties, crystallizability, nucleation, growth, and polymorph selection.

The molecular structures of the conformers from all four polymorphic forms were found to be very similar, with the three dimers (two from the α-form and one from the γ-form) also being very similar. The crystal chemistry and crystallizability behavior of the well-characterized α- and β-form PABAs are though found to be quite different, although both crystallize in the same monoclinic space group *P*2_1_/*n*. The molecular conformation deformation energy in the crystalline state and the convergence of the lattice energy for the α- and β-forms indicate that there is a higher barrier to crystallization for the β-form and also that low solution supersaturations might be needed for it to preferentially nucleate. The different intermolecular packing in PABA polymorphs leads to the different relative contributions of functional groups to the lattice energy, with the functional group analysis indicating that the α-form might be expected to be dominantly crystallized due to its strong carboxylic dimer interaction, while the dispersive π–π interactions and H-bonding interactions with the amino group become relatively more important for the β-form. The difference between the crystal chemistry of the α- and β-forms has been shown to directly correlate with the resulting different physical properties of the crystals, such as its thermal expansion, where the largest thermal expansion was found to be in the *b* axis direction for the α-form crystals with a 10 and 2 times larger thermal expansion of the *b* axis than *a* and *c* axes for α- and β-forms, respectively, correlating well with the weaker dispersive-type intermolecular interactions along the *b* axis. The predicted crystal morphology of α-PABA would be expected to be flat, lathe-like, and reasonably consistent with the experimentally observed crystal morphology, while the β-form crystallizes with a prismatic equant morphology, in good agreement with the predicted one. The weak and more isotropic π–π stacking interactions, much lower attachment energy, and much higher saturation of the surface molecule for the {101} faces for the α-form lead to lower growth rates and hence larger surface areas. In contrast, the {10–1} faces containing H-bonding interactions and being hydrophilic in nature and the capping {01–1} faces consisting of π–π stacking interactions and being more hydrophobic and dominated by the carboxylic acid dimer interactions would be expected to have higher growth rates, reflecting their higher attachment energies and lower saturations of surface molecules.

Solution dissolution measurements revealed that three solvents (EtOH, AcN, and water) exhibited less than ideal solubility with low aqueous solubility but a higher solubility in the protic solvent EtOH compared to the aprotic solvent AcN, consistent with an enhanced degree of solute/solute interactions and solute clustering within the solution structure. The interaction energies associated with the predicted solvation shell structures for 3 solvent systems, namely, EtOH, AcN, and water, were found to be consistent with their respective order associated with their measured solubilities. COSMO-RS studies indicated that the β-form would be expected to be the more likely polymorph to crystallize from aqueous solutions, while the α-form would be expected to be preferentially crystallized from most of the other solvents studied. Molecular dynamics analysis of solute cluster propensity and solute solvation energy demonstrates the importance of an aqueous solvation environment in inhibiting the α-form’s strong OH···O carboxylic acid hydrogen bond dimer. This is also supported by NMR studies. The crystallizabilities for the three solvents highlight the distinctly different behavior between these solvents with water having a much less dependence on the solution cooling rate compared to EtOH and AcN, with the crystallizability rate-limiting parameters being in the order of (kinetics-driven) EtOH > AcN > water (thermodynamics-driven). The experimental crystallizability observations also support the solvation energy calculations and the MD results; in particular, the OH···O (synthon Aα) interactions have been found to be stronger in AcN than in EtOH due to the much stronger solvation, with the hydrogen-bonded interactions being much lower than for AcN and EtOH in aqueous solutions. The nucleation mechanisms of PABA in AcN, EtOH, and water solutions were found to be mostly instantaneous, consistent with a two-step nucleation mechanism except for the lower solute concentrations, where it was progressive. Among the instantaneous cases, EtOH was found to be more instantaneous than AcN and water, which perhaps reflects their relative solubilities. Crystallization in the presence of tailor-made additives tends to lead to large increases in the MSZW but little changes in the solubility, with the nucleation pathway being found to change from instantaneous to progressive nucleation in the presence of the TMAs.

The use of NME in the solution phase within the mixed solvent (EtOH/NMe) system reveals that NMe inhibits the formation of π–π stacking in α-PABA, hence hindering the growth in the capping face direction controlled by interactions other than π–π stacking, leading to a much more isotropic morphology. Facet growth rate fittings reveal that the {01–1} capping and {10–1} prismatic faces have RIG and B&S growth mechanisms, respectively, with the former exhibiting strong π–π stacking interactions (synthon Bα) as its main contributor to the fast growth of this face {01–1} and the latter having H-bonding interactions (synthon Aα) along the growth direction involving COOH and NH_2_ functional groups as the dominant contribution, leading to slower desolvation at the {10–1} face, hence reducing its growth rate. The measured growth rates of α-PABA’s {10–1} and {01–1} faces were found to be within the same order of magnitude of those measured for the similar organic systems.

Analysis of the SAXS data at low *Q* reveals the existence in EtOH of solute clusters within the solution state that grow and develop in the supersaturation state. Complementary SAXS data at low *Q* provides evidence also for the formation of small intermolecular cluster structures (ca. 1–2 nm) within the size range that would be well matched to the overall dimensions of Aα synthon, leading to a tentative nucleation pathway for PABA crystallization from ethanol solutions being proposed.

Overall, the characteristic molecular descriptors and associated crystallographic structures of all four forms show a good consistency of their molecular descriptors with what is expected for a small-molecule pharmaceutical compound.

## 7 Some Future Perspectives

The transfer of the fundamental molecular-scale understanding of PABA crystallization into crystallization process design has been quite limited to date, and this may well serve the basis of significant future work on this system. For example, combining nucleation kinetics, facet growth mechanisms with population balance modeling could lead to a better morphological control of the product form and a more reliable scale-up of crystallization processes, hence enabling their digital design and optimization.

In this respect, the design of the mixed suspension mixed product removal (MSMPR) cascade has been found to be able to identify the operational window of the process variables, stage temperature, and residence time for achieving the stringent polymorph purity and high yield of PABA crystals.^87^ In this, β-PABA was identified as the dominant form at the steady state in single-stage MSMPR below the transition temperature of 15 °C, while α-PABA was found to be dominant when operating above that temperature. To control the polymorphism from β-PABA to α-PABA at the 5 °C stage, a two-stage MSMPR was developed, with the first stage operating at the thermodynamic control regime (30 °C) for the α polymorph and the second stage to increase the secondary nucleation and mass deposition rates of the α-PABA crystals in the 5 °C stage, hence delivering a high yield.^87^

A morphological population balance modeling approach,^88^ integrating crystal morphology and facet growth kinetics with multidimensional population balance, has been applied to simulate the batch crystallization of α-PABA from EtOH solutions as a function of the crystallization environment including the cooling rate, seeding temperature (supersaturation), and seed conditions (loading, size and shape, and broken seeds). From the examination of the evolution of the crystal shape/size and their distributions, it was found that a higher seed loading produced smaller and less needle-like crystals with similar yields, hence potentially being an important parameter for process control. The fracture surfaces of broken seeds, mimicking the milled seed conditions in practice, were found to grow fast, hence rapidly disappearing from the external crystal morphology.

Finally, much of the work has focused on the crystallization science underpinning the formation of the α-form, and, in the future, understanding on the behavior of the other forms can be expected through studies on their solution, solid-state, and surface properties and how such a behavior is mediated by the molecular nature of the solvents. Such key understandings are not only generic, but also arguably a vital resource for the preparation of the high-quality crystalline materials needed for the future, notably high purity, narrow size distribution, and high crystal lattice perfection. Hence, the broad concepts encompassed here can be expected to impact directly on the digital design of structured particulate products together with the crystallization processes^89^ needed for their manufacture. This review has hopefully demonstrated the utility of the PABA crystallization as a model system for the examination of crystal science underpinning the preparation of a representative molecular material system. Further studies can be expected to build on and enhance the work carried out to date.

## List of Symbols and Abbreviations

*a*, *b*, *c*: crystal unit cell parameters (Å)
D···A: bond length between the donor and the acceptor (Å)
D-H: bond length between the donor and hydrogen (Å)
D-H···A: angle of donor–hydrogen–acceptor (Å)
H···A: hydrogen bond length between the acceptor and hydrogen (Å)
*hkl*: crystal face index
*I*_(q)_: scattered intensity
MSZW: metastable zone width (°C)
*q*: solution cooling rate (m s^–1^)
*Q*: scattering vector (nm^–1^)
RMSD: root mean-squared deviation
*r**: molecular cluster size (nm)
*R* ^*2*^: coefficient of determination
*T*_c_: crystallization onset point (critical temperature) (°C)
*T*_e_: equilibrium solubility temperature (°C)
*u*_c_: relative critical undercooling
*Z/Z*′: number of molecules in the asymmetric cell/unit cell
β: crystal unit cell parameter (deg)
Δ*H*_diss_: enthalpy of dissolution (kcal mol^–1^)
Δ*T*_c_: critical undercooling (°C)
γ_eff_: interfacial tension (mJ m^–2^)
σ: relative supersaturation
ξ: saturation of the surface molecule (anisotropy factor)
A: H-bond acceptor
AcN: acetonitrile
AMBA: 4-amino-3-methoxybenzoic acid
ANBA: 4-amino-3-nitrobenzoic acid
API: active pharmaceutical ingredient
Aα, Aβ: synthon A of α-form, synthon A of β-form
B&S: birth and spread growth mechanism
D: H-bond donor
DFT: density functional theory
DMSO: dimethyl sulfoxide
EtOAc: ethyl acetate
EtOH: ethanol
FTIR: Fourier transform infrared spectroscopy
H2H: head-to-head
H2T: head-to-tail
H-bond or HB: hydrogen bond
IR: infrared
M1, M2: molecule 1, molecule 2
MD: molecular dynamics
MeOH: methanol
MSMPR: mixed suspension mixed product removal
NMe: nitromethane
NMR: nuclear magnetic resonance
PABA: *p*-aminobenzoic acid
RIG: rough interface growth mechanism
SAXS: small-angle X-ray scattering
TMA: tailor-made additive
XRD: X-ray diffraction
